# Endothelial cell activation on 3D-matrices derived from PDGF-BB-stimulated fibroblasts is mediated by Snail1

**DOI:** 10.1038/s41389-018-0085-z

**Published:** 2018-09-24

**Authors:** Alberto Herrera, Mercedes Herrera, Natalia Guerra-Perez, Cristina Galindo-Pumariño, María Jesús Larriba, Vanesa García-Barberán, Beatriz Gil, Sara Giménez-Moyano, Reyes Ferreiro-Monteagudo, Pilar Veguillas, Antonio Candia, Raúl Peña, Jesús Pinto, Mª Laura García-Bermejo, Alberto Muñoz, Antonio García de Herreros, Félix Bonilla, Alfredo Carrato, Cristina Peña

**Affiliations:** 1 Department of Medical Oncology, Hospital Universitario Puerta de Hierro de Majadahonda, Majadahonda, Madrid Spain; 2 Medical Oncology Department, Instituto Ramón y Cajal de Investigación Sanitaria (IRYCIS), Madrid, Spain; 3 Instituto de Investigaciones Biomédicas Alberto Sols, Consejo Superior de Investigaciones Científicas-Universidad Autónoma de Madrid, CIBERONC, Madrid Spain; 4 Laboratorio de Oncología Traslacional y Nuevas Terapias. Instituto de Investigación i+12, Madrid, Spain; 5 Biomarkers and Therapeutic Targets Lab, Pathology Department, Instituto Ramón y Cajal de Investigación Sanitaria (IRYCIS), Madrid, Spain; 6 Surgery Department, Hospital Universitario de Guadalajara, Guadalajara, Spain; 7 Pathology Department, Hospital Universitario de Guadalajara, Guadalajara, Spain; 8 Programa de Recerca en Càncer, Institut Hospital del Mar d’Investigacions Mèdiques, Barcelona, Spain; 9 Pathology Department, Virgen de la Concha Hospital, Zamora, Castilla y León Spain; 10 Centro de Estudios Biosanitarios, Madrid, Spain; 11 Medical Oncology Department, Ramon y Cajal University Hospital, IRYCIS, CIBERONC, Alcala University, Madrid, Spain; 12 Present Address: Department of Oncology and Pathology, Karolinska Institutet, Stockholm, Sweden; 13 Present Address: Laboratory of Molecular Oncology, IIS Hospital Clínico San Carlos, CIBERONC, Madrid Spain; 14 Present Address: Medical Oncology Department, Instituto Ramón y Cajal de Investigación Sanitaria (IRYCIS), CIBERONC, Madrid Spain

## Abstract

Carcinomas, such as colon cancer, initiate their invasion by rescuing the innate plasticity of both epithelial cells and stromal cells. Although Snail is a transcriptional factor involved in the Epithelial-Mesenchymal Transition, in recent years, many studies have also identified the major role of Snail in the activation of Cancer-Associated Fibroblast (CAF) cells and the remodeling of the extracellular matrix. In CAFs, Platelet-derived growth factor (PDGF) receptor signaling is a major functional determinant. High expression of both SNAI1 and PDGF receptors is associated with poor prognosis in cancer patients, but the mechanism(s) that underlie these connections are not understood. In this study, we demonstrate that PDGF-activated fibroblasts stimulate extracellular matrix (ECM) fiber remodeling and deposition. Furthermore, we describe how SNAI1, through the FAK pathway, is a necessary factor for ECM fiber organization. The parallel-oriented fibers are used by endothelial cells as “tracks”, facilitating their activation and the creation of tubular structures mimicking in vivo capillary formation. Accordingly, Snail1 expression in fibroblasts was required for the co-adjuvant effect of these cells on matrix remodeling and neoangiogenesis when co-xenografted in nude mice. Finally, in tumor samples from colorectal cancer patients a direct association between stromal SNAI1 expression and the endothelial marker CD34 was observed. In summary, our results advance the understanding of PDGF/SNAI1-activated CAFs in matrix remodeling and angiogenesis stimulation.

## Introduction

Emerging evidences indicate that human carcinomas often have significant stromal reactions, characterized by the existence of stromal cells and extracellular matrix proteins^1^. In solid tumors, including primary and metastatic colorectal cancer (CRC), fibroblasts are the main component of tumor stroma, receiving various names, such as Cancer-Associated Fibroblasts (CAFs)^1^. CAF populations are heterogeneous^2,3^ and directly promote tumor growth and progression throughout the induction of stem cell properties, tumor cell motility and implantation, but also enhance angiogenesis, inflammation and ECM remodeling^4^.

CAFs are characterized by the upregulation of proteins, such as α-SMA, fibroblast specific protein 1 (FSP1), fibroblast activation protein (FAP) and platelet-derived growth factor receptors (PDGFR)-α/β^5,6^. High stromal expression or activation of PDGFR-β is associated with poor prognosis in breast, prostate and gastrointestinal tumors^7–10^. The involvement of PDGF isoforms in both autocrine and paracrine stimulation of tumor growth has been extensively studied^11,12^. PDGF-BB expression, by tumor epithelial cells or by endothelial cells (ECs), enhances angiogenesis, by recruiting pericytes to neovessels, and promotes an activated phenotype on fibroblasts^13–16^.

Snail1, a zinc finger transcriptional factor, is key in the initiation of epithelial-mesenchymal transition (EMT)^17^. Although adult fibroblasts normally do not express Snail1^18^, there are some situations in which Snail1 protein is detected in these cells, such as wound healing or cancer progression^18–21^.

As mentioned above, PDGF-BB induces an activated state in fibroblasts^13,22^, but the molecular pathways are poorly defined. We proposed Snail1 as a marker of activated fibroblasts^21^. Snail1-expressing fibroblasts promote ECM deposition accompanied by ECM degradation, and increase the stiffness and orientation of ECM fibers^19,23–25^. In addition, ECM is a major regulator of vasculogenesis and angiogenesis: it is a physical scaffold that controls endothelial cell activity through chemical and mechanical signals^26^. Here, we demonstrate that PDGF-BB stimulates Snail1 expression in fibroblasts, in a FAK pathway-dependent manner, resulting in matrix remodeling. 3D-derived matrices from Snail1-expressing fibroblasts induce tubulogenesis in endothelial cells. In animal models, xenografted tumors composed by colon tumor cells and Snail1 KO fibroblasts showed reduced ability for neoangiogenesis with respect to Snail1 wild-type fibroblasts. Moreover, we observed a direct association between Snail1 stromal expression and angiogenesis in human colon cancer patients. These findings reveal a new role for Snail1-expressing fibroblasts and the PDGF pathway in tumor angiogenesis.

## Results

### PDGF stimulates growth and extracellular matrix production of fibroblasts

**Fig. 1:**
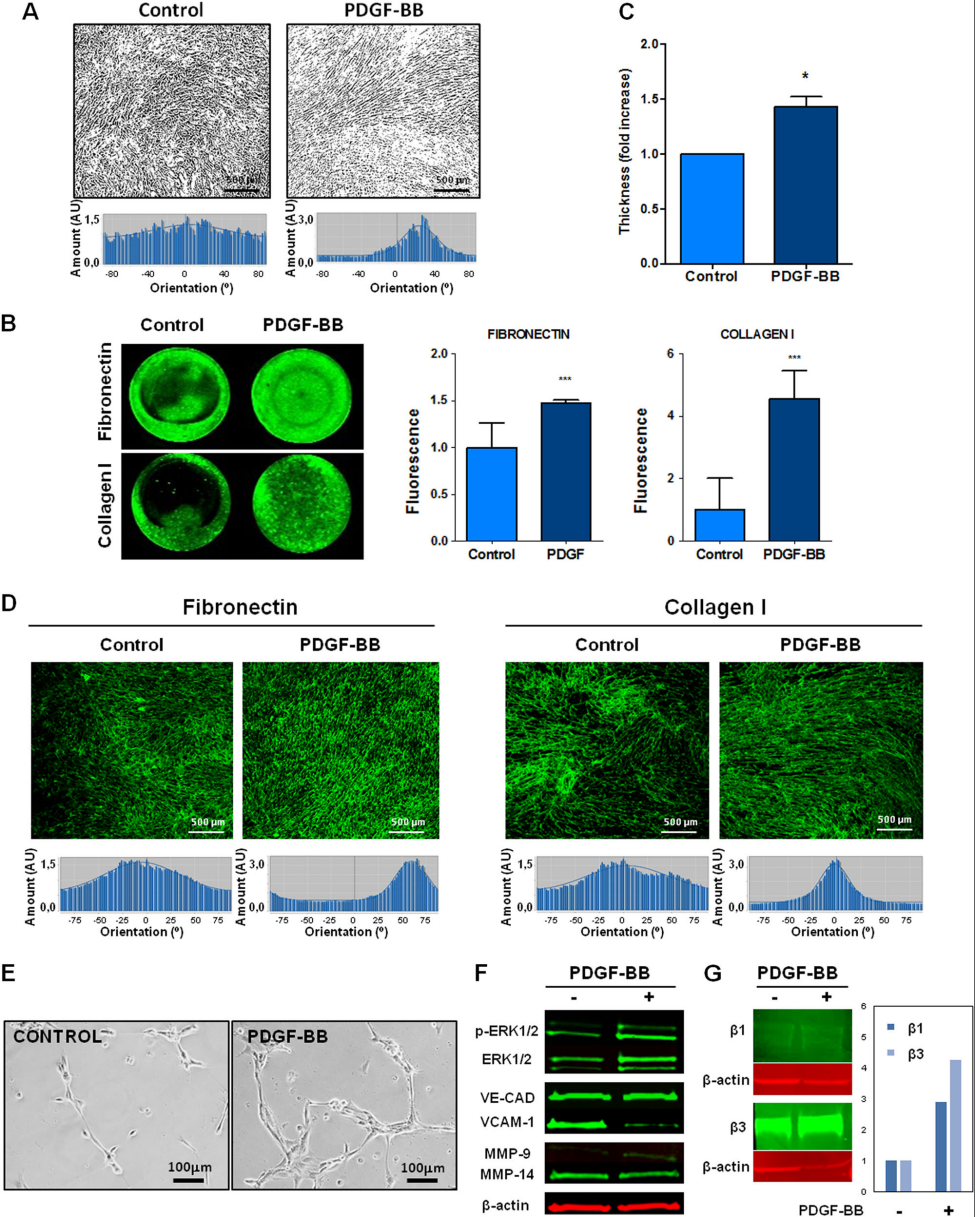
PDGF-stimulated fibroblasts enhance thicker and anisotropic extracellular matrix, which promotes endothelial cell activation. **a** Binary images representative of the cellular orientation of the BJ-hTERT fibroblasts treated or not with PDGF-BB (above) and directionality histograms (below) calculated with the Image J software which represent the frequency of distribution of cell angles (centered on the 0 ° angle). **b** Increase in protein expression of extracellular proteins such as fibronectin and Collagen I in PDGF-stimulated fibroblasts derived from ECM. **c** Increase of ECM thickness, determined by confocal microscopy, in those ECMs derived from PDGF-stimulated fibroblasts. **d** Increase in protein and organization of extracellular proteins such as fibronectin and Collagen I in PDGF-stimulated fibroblasts derived from ECM. Under the image its represented the directionality histograms calculated with the Image J software which represent the frequency of distribution of protein fibers angles (centered on the 0 ° angle). **e**, **f** Endothelial cell activation was observed by formation of capillary-like structures on matrices derived from PDGF-stimulated fibroblasts (**e**) and increased expression of angiogenesis-related markers (**f**). **g** Increase expression of β1 and β3 integrins subunits in HUVECs from PDGF-derived matrices. *p value < 0,05; ***p value < 0,001. All results are derived from 2–4 independent experiments, each performed in duplicate

To study the possible influence of PDGF ligand on ECM characteristics, BJ-hTERT fibroblasts were incubated with or without exogenous PDGF; the cells were supplemented with ascorbic acid to enhance matrix production. After PDGF treatment, fibroblasts displayed a more aligned phenotype (Fig. 1a).

3-D matrices derived from PDGF-stimulated fibroblasts were decellularized and stained for Collagen I and Fibronectin to study matrix composition and structural changes. Collagen I and Fibronectin protein expression increased in matrices derived from PDGF-stimulated fibroblasts (Fig. 1b). Consequently, matrix thickness was increased by PDGF-BB stimulation, as measured by Confocal Microscopy (Fig. 1c). Moreover, Fibronectin and Collagen I fibers revealed a parallel pattern, determined by Directionality Histograms, in PDGF-stimulated fibroblasts, as shown in Fig. 1d. We previously described the involvement of the p65 subunit of NF-κB in activating fibronectin transcription^25^. Currently, our results showed an increase of p65 phosphorylation in BJ-hTERT fibroblasts after PDGFBB stimulation (Sup. Fig. 1). The observed high degree of organization of ECM fibers induced by PDGF is not due to the number of BJ-hTERT fibroblasts, since this number was not different at the end of the experiment (Sup. Fig. 2).

A previous study of our group determined the gene expression profile of control and PDGF-stimulated BJhTERT fibroblasts (GEO Series accession number: GSE40720). We re-analyzed these data focusing on the protein related with the composition, organization and remodeling of the ECM. Gene Set Enrichment Analysis (GSEA) was done using the Molecular Signatures Database (MSigDB) which is a collection of annotated gene sets for use with GSEA software. (http://software.broadinstitute.org/gsea/msigdb/collections.jsp). Our analysis computed overlaps between our gene set and gene sets in MSigDB (we included gene sets derived from the KEGG and Reactome pathway databases and the hallmark gene set database). The analysis shows important gene sets involved, with FDR q-value below 0.05. Interestingly, the Extracellular Matrix Organization and the Collagen Formation is observed as one of the main biological processes involved. Similarly, angiogenesis regulation is also observed as a candidate hallmark to be affected by the deregulated genes (Sup. Table 1).

### Derived Matrices from PDGF-stimulated fibroblasts enhance the activation and tubulogenesis of HUVECs

To study the role of the extracellular matrix on the endothelial cell functions, human endothelial HUVEC cells were seeded over decellularized matrices derived from PDGF-stimulated or non-stimulated BJ-hTERT fibroblasts. As shown in Sup, Fig. 3, HUVEC cells do not express PDGF-β receptor and do not tyrosine phosphorylate Akt following addition of exogenous PDGF. Interestingly, HUVECs seeded on matrices from PDGF-stimulated fibroblasts showed more defined networks of capillary-like structures and anastomosing cords in cells (commonly known as tube formations), than in HUVECs seeded on matrices from non-stimulated fibroblasts (Fig. 1e).

Angiogenesis-related markers (Fig. 1f), including phospho-ERK1/2 and MMP-9, increased in HUVECs from PDGF-derived matrices. MT1-MMP also increased slightly in these matrices, while VCAM-1 decreased and no changes in VE-Cadherin were detected. In addition, an increase expression of β1 and β3 integrins subunits, related with the attachment of the endothelial cells to the ECM for neoangiogenesis, was observed in HUVECs from PDGF-derived matrices (Fig. 1g).

Taken together, these data demonstrate that 3D-ECMs derived from PDGF-stimulated fibroblasts stimulate HUVEC activation and attachment to ECM.

### Endothelial cell activation is blocked by the inhibition of Collagen I fiber organization in fibroblasts-derived matrices

**Fig. 2:**
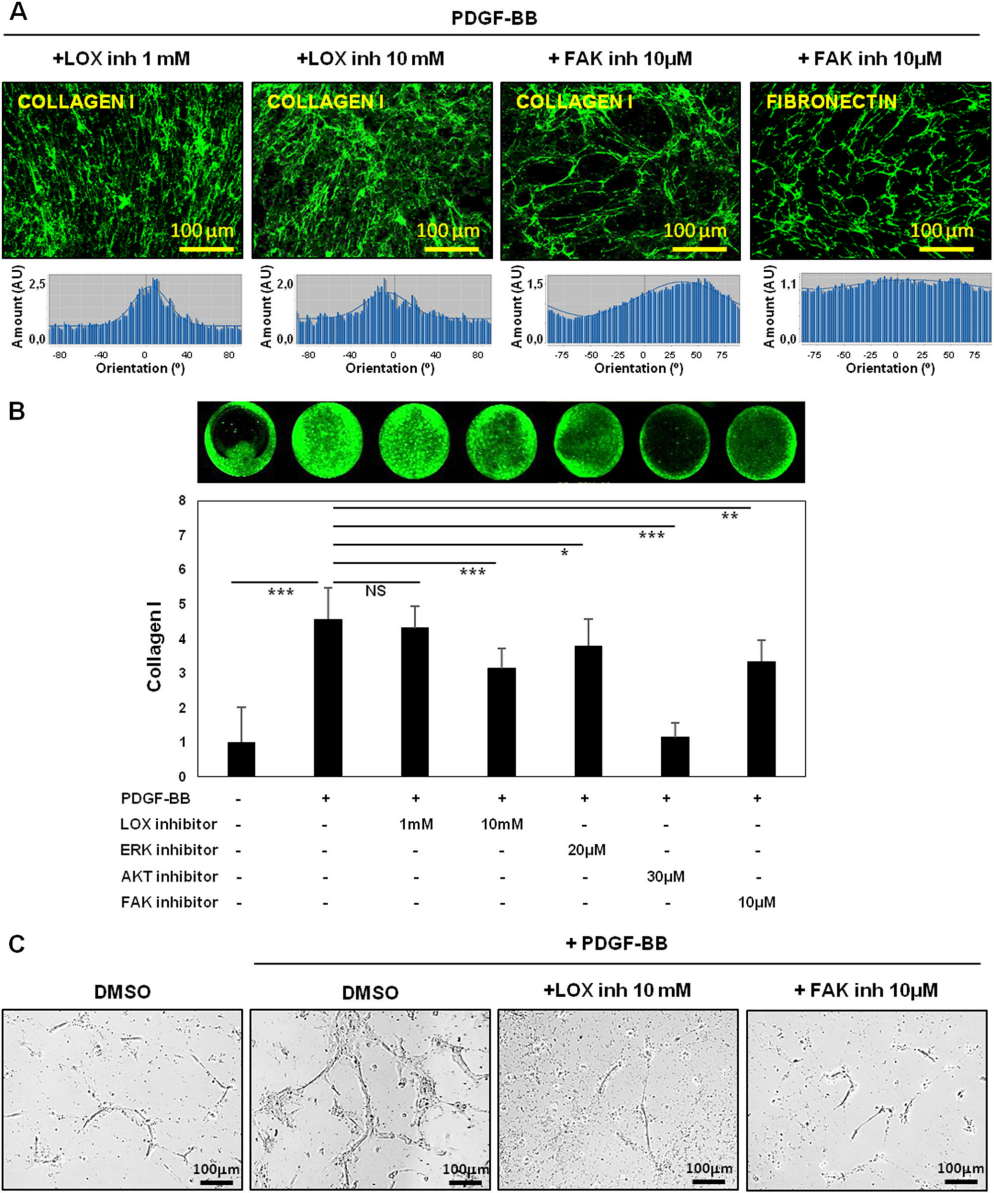
Fibroblast alignment, matrix remodeling and endothelial cell activation are dependent on Collagen I organization and FAK. **a**, **b** Collagen I organization (**a**) and deposition (**b**) were blocked by increased concentrations of LOX or FAK inhibitors. Directionality histograms, calculated with the Image J software, represent the frequency of distribution of protein fibers angles (centered on the 0 ° angle). **c** Full inhibition of endothelial tubulogenesis in matrices derived from LOX or FAK inhibitor-treated fibroblasts. *p value < 0,05; **p value < 0,01; ***p value < 0,001. All results are derived from 2–4 independent experiments, each performed in duplicate

To investigate whether fiber alignment was responsible for the tubulogenesis process on 3D-derived matrices, we proceed to inhibit Collagen fiber organization using a pharmacological approach. Fist we used a competitive inhibitor (beta-aminopropionitrile, BAPN) of the activity of the Collagen-crosslinker enzyme, lysyl oxidase (LOX)^27^. In line with previous data^19^, the effects of PDGF-BB on Collagen I organization and deposition were blocked by BAPN (Fig. 2a, b, respectively) although this compound did not affect fibroblast growth or organization (Sup. Fig. 4a). Neither Fibronectin fiber disposition nor the amount were affected by BAPN (Sup. Fig. 4B and 4C). Predictably, no changes in LOX protein were observed, since BAPN functions as an inhibitor of LOX activity (Sup. Fig. 4D).

Interestingly, the PDGF-dependent up-regulation of tubulogenesis was reverted when endothelial cells were seeded on BAPN-treated 3D matrices, demonstrating the pivotal role of fiber organization in this process (Fig. 2c).

Since PDGF signals through Erk, PI3K/Akt and FAK pathways, we also used inhibitors for these pathways to assess a possible role of these proteins in the regulation of the ECM changes. All these compounds specifically inhibited the PDGF-stimulated phosphorylation of its target (Sup. Fig. 5).

Collagen I deposition was completely reverted by the Akt inhibitor (LY294002) (Fig. 2b) whereas the FAK inhibitor (PF573228) showed a more prominent effect on both fibroblast organization and matrix fiber orientation (Sup. Fig. 6A and Fig. 2a, b). On the other hand, fibroblast growth and matrix fiber orientation were not affected by the ERK inhibitor (UO126), that only caused a slight reduction in Collagen I deposition (Sup. Fig. 6A, 6B and 6C). Accordingly, HUVEC tubulogenesis was completely inhibited in matrices derived from FAK inhibitor-treated fibroblasts (Fig. 2c).

Taken together, these experiments identify the FAK pathway as an important mediator of PDGF-dependent effects in matrix deposition and alignment by fibroblasts, consequently this pathway inhibition prevent endothelial cell activation.

### Fibroblast alignment, matrix remodeling and enhanced tubulogenesis by PDGF are dependent on Snail1-fibroblast expression

We recently described Snail1 as a potential marker of activated fibroblasts with paracrine-derived pro-tumorigenic effects on colon cancer cells^21^. We checked the effect of the ERK1/2, PI3K/Akt and FAK inhibitors on Snail1 protein expression in PDGF-stimulated fibroblasts. Snail1 was induced at serum-free levels in PDGF-treated fibroblasts, with maximum expression at 24 h, together with increased α-SMA expression, the actin isoform involved in ECM contraction and remodeling (Sup. Fig. 7A and 7B). Moreover, Snail1 nuclear translocation was also observed in PDGF-treated fibroblasts (Sup. Fig. 7C). α-SMA was remarkably down-regulated by Akt and FAK inhibitors (Sup. Fig. 7d); in contrast Snail1 expression was only sensitive to the FAK inhibitor (Sup. Fig. 7C and D). Therefore, the induction in Snail1 expression in fibroblasts treated with PDGF is dependent on the FAK pathway.

**Fig. 3:**
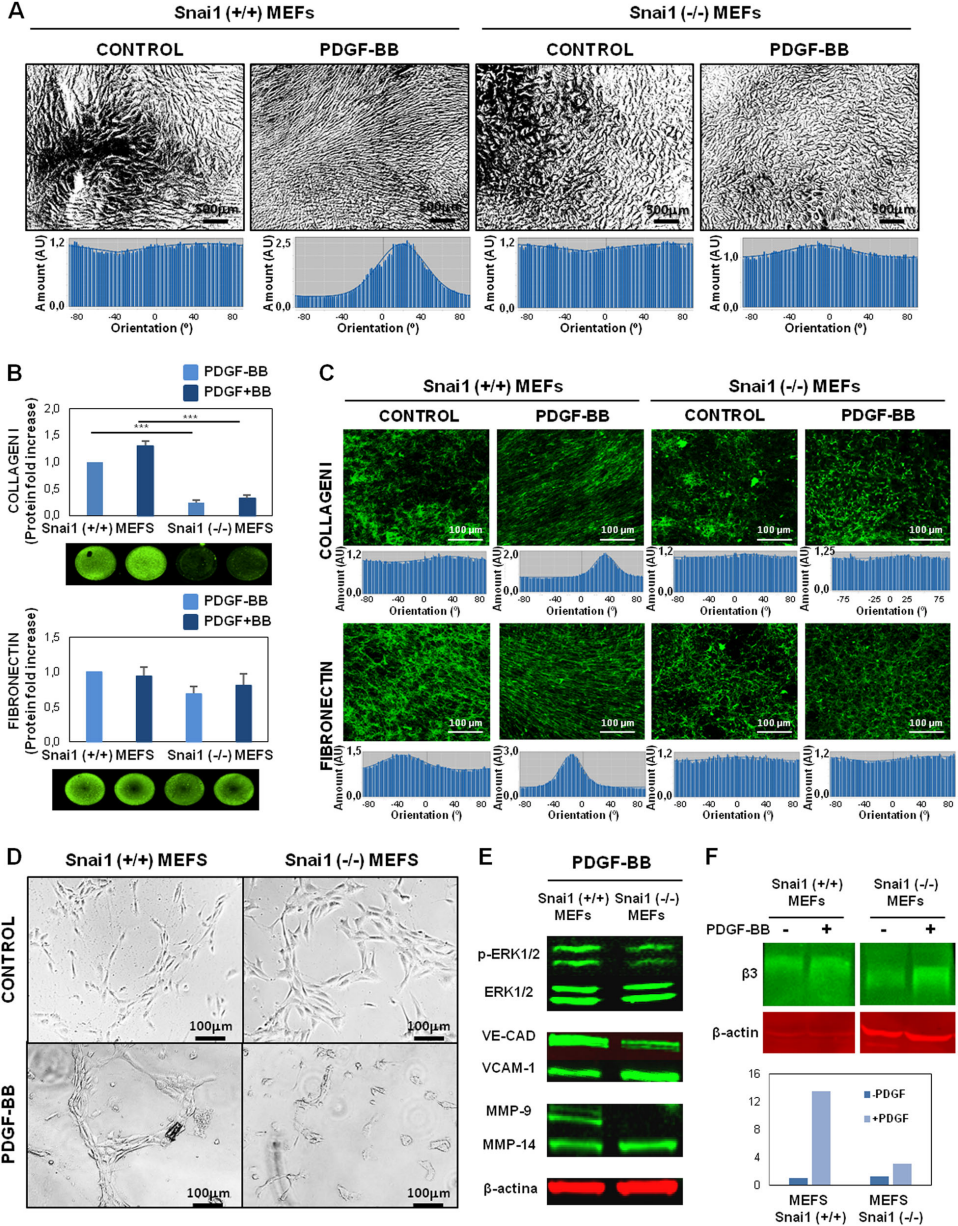
Snail regulates fibroblast alignment, matrix remodeling and tubulogenesis in response to PDGF. **a** SNAI 1 expression is required for alignment of fibroblasts dependent on PDGF ligand. Below the representative images the directionality histograms, calculated with the Image J software, show the frequency of distribution of cell angles (centered on the 0 ° angle). **b**, **c** Increase in ECM fiber deposition (**b**) and Organization (**c**) in matrices derived from PDGF-stimulated fibroblasts were dependent on SNAI1 expression. Under the image its represented the directionality histograms calculated with the Image J software, representing the frequency of distribution of protein fibers angles (centered on the 0° angle). **d**, **e** Formation of capillary-like structures on matrices derived from PDGF-stimulated fibroblasts (**d**) and increased expression of angiogenesis-related markers (**e**) are blocked in SNAI1 KO fibroblasts. **f** Down-regulation of β3 integrins in HUVECs from SNAI1 KO fibroblasts matrices. All results are derived from 2–4 independent experiments, each performed in duplicate

Furthermore, Snail1 has previously been described as modifying the expression of some ECM proteins, including fibronectin and Collagen I^25,28–31^. In this way, 1.BR3G human fibroblasts with ectopic SNAI1 overexpression were incubated with PDGF and the expression of different ECM proteins were analyzed by the Array Human Extracellular Matrix and Adhesion Molecules. Interestingly, as it is shown in Sup. Table 2, a synergic effect of SNAI1 overexpression and PDGF stimulation is observed regarding the regulation of several ECM related proteins, including structural proteins as different types of Collagen, Fibronectin, Laminins; ECM adherents proteins as Integrins subunits; Secreted proteins as Metalloproteinases, TGFβ, TIMP2.

To deeply study the possible effects of Snail1 expression on ECM, 3-D matrices were generated from Mouse Embryonic Fibroblasts (MEFs) either wild-type (wt) or KO for SNAI1 under PDGF stimulation. A greater alignment of cells was shown in wt MEFs on PDGF stimulation (Fig. 3a), as was previously observed in BJ-hTERT cells. In contrast, alignment of fibroblast cells was not observed when Snail1 was depleted in fibroblasts, independently of PDGF stimulation (Fig. 3a).

Decellularized 3D-ECMs generated by KO MEFs showed fewer Fibronectin and specifically Collagen I than matrices produced by wt MEFs (Fig. 3b). In parallel, a decrease of p65 phosphorilation was observed in KO MEFs regarding wt MEFs (Sup. Fig. 8). Moreover, wt MEFS upon PDGF stimulation produced more Collagen I, whereas no changes in the Collagen I amount were observed in KO MEFs. Directionality histograms showed that wt MEFs treated with PDGF exhibited a high degree of both Fibronectin and Collagen I fiber organization (Fig. 3c). In contrast, PDGF failed to reorganize ECM fibers in KO MEF-derived matrices (Fig. 3c).

After fibroblast removal, HUVECs were seeded on 3-D derived matrices to study the angiogenic switch and tubulogenesis. Enhanced tubulogenesis was observed in matrices derived from wt MEFs treated with PDGF, while these capillary-like structures were not observed in those matrices derived from non-stimulated wt. Addition of PDGF to Snail1 KO MEFs did not increase HUVEC tubulogenesis effects found in HUVECs seeded on Snail1 KO MEF-derived matrices, regardless of PDGF stimulation (Fig. 3d).

In accordance to this data, matrices derived from PDGF-stimulated fibroblasts significantly modify angiogenic and ECM attachment markers of endothelial cells in a Snail1-dependent manner. Thus, a decrease in endothelial cell activation markers (pERK1/2, VE-Cadherin, MMP-9) and slightly higher levels of VCAM1 were observed in HUVEC cells seeded on Snail1 KO MEF-derived matrices (Fig. 3e). The increase expression of β3 integrins subunits was also observed in HUVEC cells seeded on wt MEF-derived matrices with PDGF treatment. In contrast, a down-regulation of β3 integrins was observed when Snail1 was depleted in fibroblasts, and PDGF stimulation only produced a vaguely increase of them (Fig. 3f).

Taken together, these analyses demonstrated that depletion of Snail1 from fibroblasts blocked their ability to enhance endothelial tubulogenesis in a PDGF/FAK-dependent manner.

### Snail1 expressing fibroblasts enhances invasion and proliferation of HUVECs

Angiogenesis requires ECM remodeling to allow the migration and invasion of endothelial cells into surrounding stroma. To investigate further the effect of Snail-expressing fibroblasts on the invasion of endothelial cells, we developed an organotypic co-culture system in which control or Snail1 KO MEFs were embedded in type I Collagen gel plus PDGF stimulation, and HUVECs were seeded at the top of the gel.

**Fig. 4:**
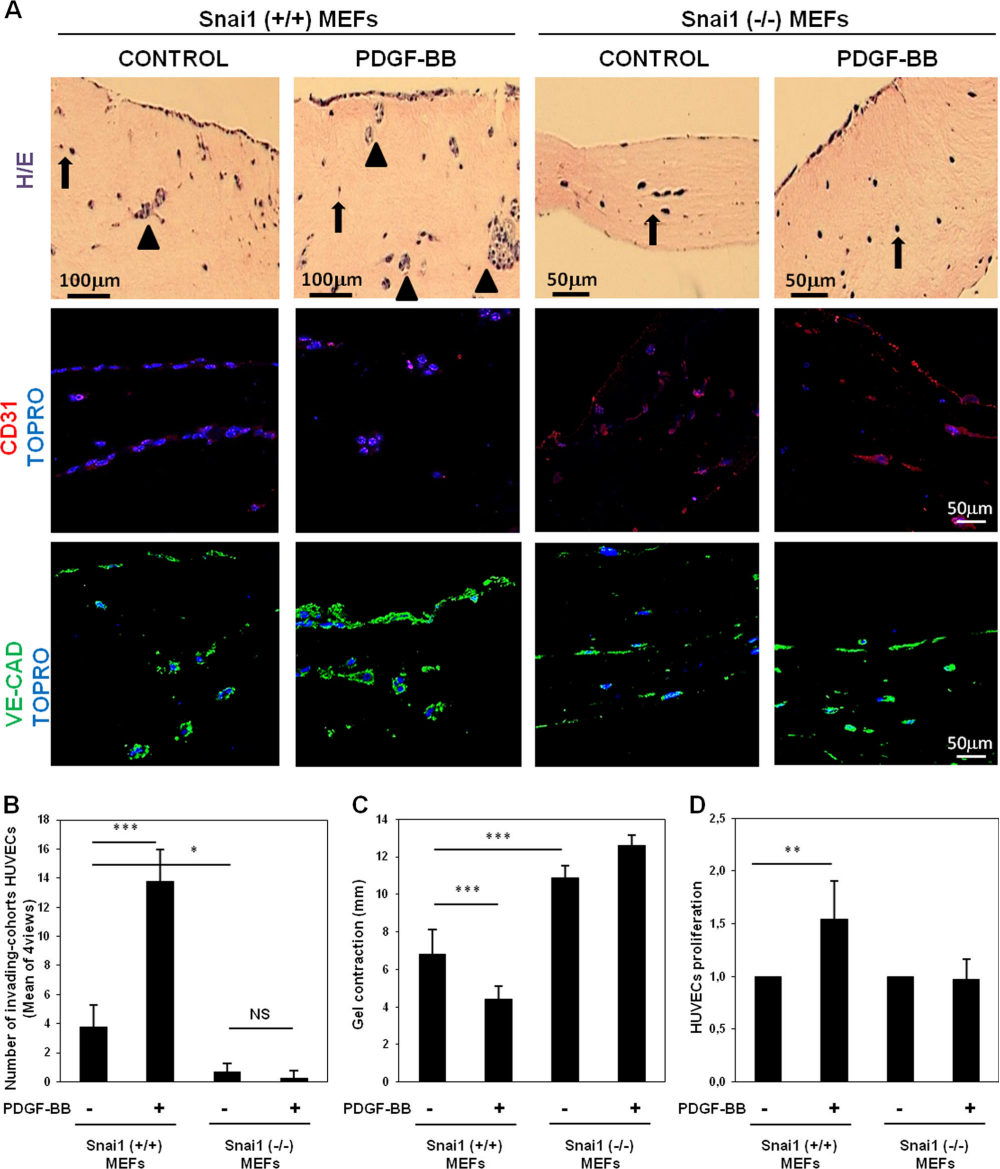
Invasion and proliferation of HUVECs are dependent on SNAI1-expressing fibroblasts. **a** Snail1 KO MEFs gels had fewer invading endothelial cells as both individual invasion (arrows) and collective cohorts (arrowheads) for Snail1 wt MEFs. Moreover, PDGF treatment enhances collective cohort invasion in HUVEC cells seeded on gels derived from Snail1 wt MEFs, but not in those derived from Snail1 ko MEFs. **b** Quantification of collective cohorts of invading HUVEC cells. **c** Deletion of Snail1 in ko MEFs avoided Collagen I gel contraction even under PDGF treatment. **d** Snail1 deletion blocked the increase of endothelial cell proliferation in cells seeded on ECM derived from PDGF-stimulated fibroblasts. *p value < 0,05; **p value < 0,01; ***p value < 0,001. All results are derived from 2–4 independent experiments, each performed in duplicate

Collagen gels including Snail1 wt MEFs showed abundant invasive endothelial cells, with individual (arrows) and collective cohort invasion (arrowheads) (Fig. 4a, b). In contrast, when Snail1 KO MEFs were included only a few individual invading cells or small cell groups were observed (Fig. 4a, b).

Furthermore, PDGF addition to gels with wt MEFs induced a higher HUVEC invasion as collective cohort (Fig. 4a, b). No PDGF effects were observed on HUVEC invasion seeded on matrices with Snail1 KO fibroblasts (Fig. 4a, b).

The capability to contract Collagen gels is a typical trait of activated fibroblast^3^. We evaluated collagen contraction in gels containing WT or Snail1 KO MEFs. After 6 days co-culture, the gel diameter was measured before the air-liquid co-culture phase and invasion of HUVECs. Collagen I gels were contracted to their maximum level, reaching 4.40 ± 0.70 mm diameter in PDGF-treated gels with control fibroblasts *vs*. 6.80 ± 1.32 mm diameter in non-treated gels with control fibroblasts. However, no clear changes in gel contraction were found in gels from non-treated (10.88 ± 0.64 mm) or PDGF-treated Snail1 KO MEFs (12.60 ± 0.55) (Fig. 4c).

Another essential step in angiogenesis is the proliferation of endothelial cells. Therefore, we next studied whether 3D-derived matrices from PDGF-stimulated fibroblasts enhance endothelial cell proliferation in a Snail1-dependent way. Green fluorescent-labeled HUVECs cultured on decellularized 3D-ECMs generated by PDGF-treated Snail wt MEFs showed a higher proliferation rate than non-treated control MEFs or Snail1 KO MEFs, regardless of the growth factor stimulation (Fig. 4d).

These results reveal the role of Snail1-activated fibroblasts in endothelial cell angiogenesis, through mechanisms involving activation, invasion and proliferation.

### PDGF and SNAI1 also control matrix remodeling in CAFs

**Fig. 5:**
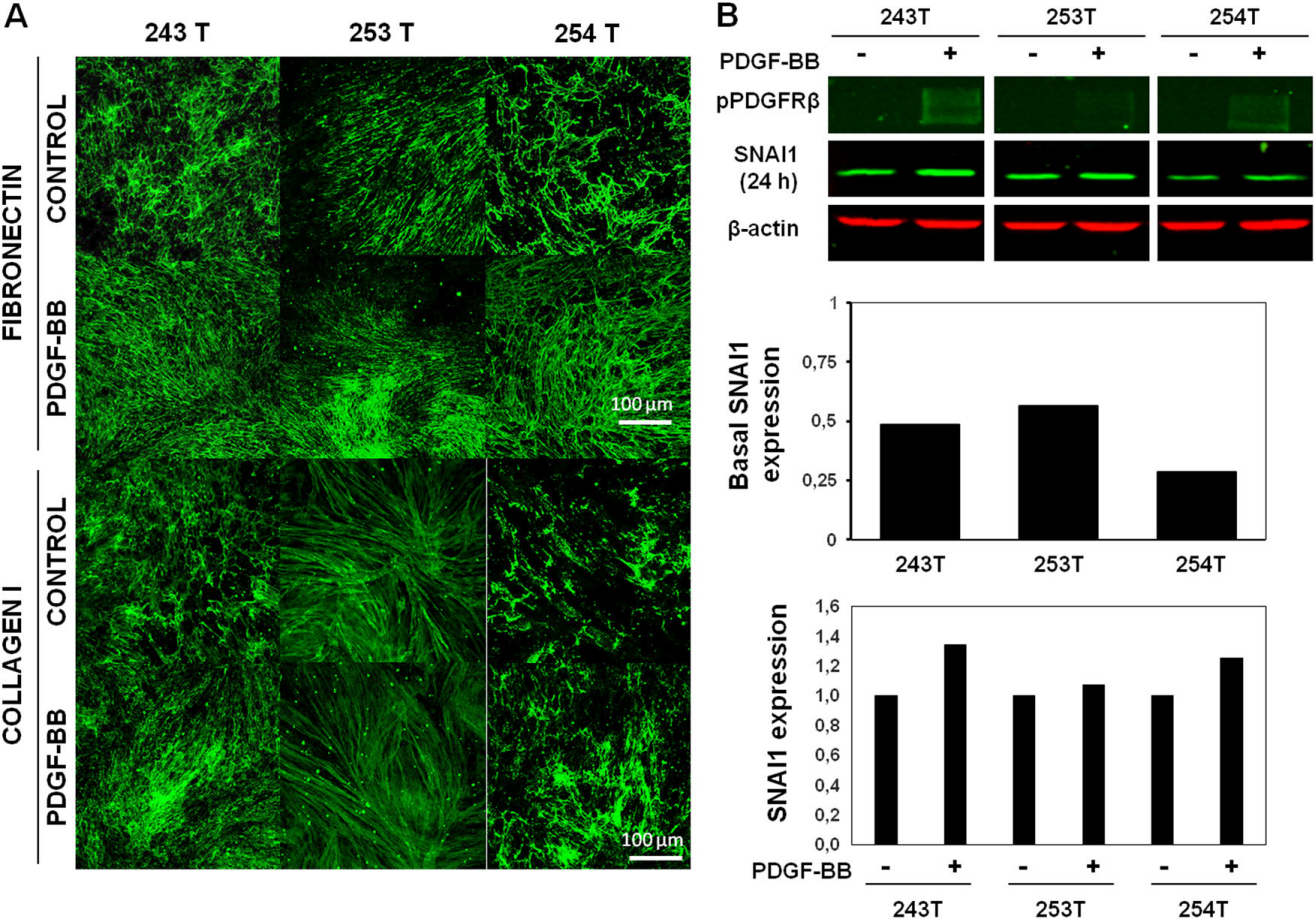
PDGFR-β/Snail1-fibroblast expression as mediators of Matrix remodeling in cancer-associated fibroblasts. **a** PDGF-treated CAFs showed greater fiber orientation in derived ECM. **b** PDGFR-β phosphorylation levels were associated with SNAI1 expression levels in primary CAFs

To verify the previous results about PDGF-fibroblast activation and matrix remodeling, we took advantage of our primary fibroblast isolation method as an *ex vivo* approach^32^. We first compared ECM alignment in normal fibroblasts (NFs) and CAFs from 3 colorectal patients. The images revealed an increase in ECM organization in CAFs, as seen after the analysis of Collagen I and Fibronectin fibers by immunofluorescence (Sup. Fig. 9). PDGF treatment of CAFs from 3 colorectal patients showed that CAFs generated 3D-ECMs with a higher degree of fiber orientation than non-treated CAFs did (Fig. 5a). When we checked SNAI1 expression and PDGFR-β phosphorylation, different levels of response to the ligand were observed in CAFs. Interestingly, basal levels of SNAI1 (without PDGF stimulation) were associated with organization of ECM fibers and the PDGFR-β phosphorylation levels correlated directly with Snail1 expression levels. In addition, CAFs with higher levels of Snail1 showed an anisotropic organization of ECM fibers (Fig. 5b).

### Snail1-expressing fibroblasts stimulate angiogenesis in the colon tumor xenograft model

To study the Snail1-expressing effects of fibroblasts on tumor angiogenesis in an in vivo tumor model, a xenograft colon cancer model was generated. HT29-M6 human colon tumor cells were subcutaneously co-injected with MEFs, either wild type or Snail1 KO. HT-29 M6 were also injected alone.

**Fig. 6:**
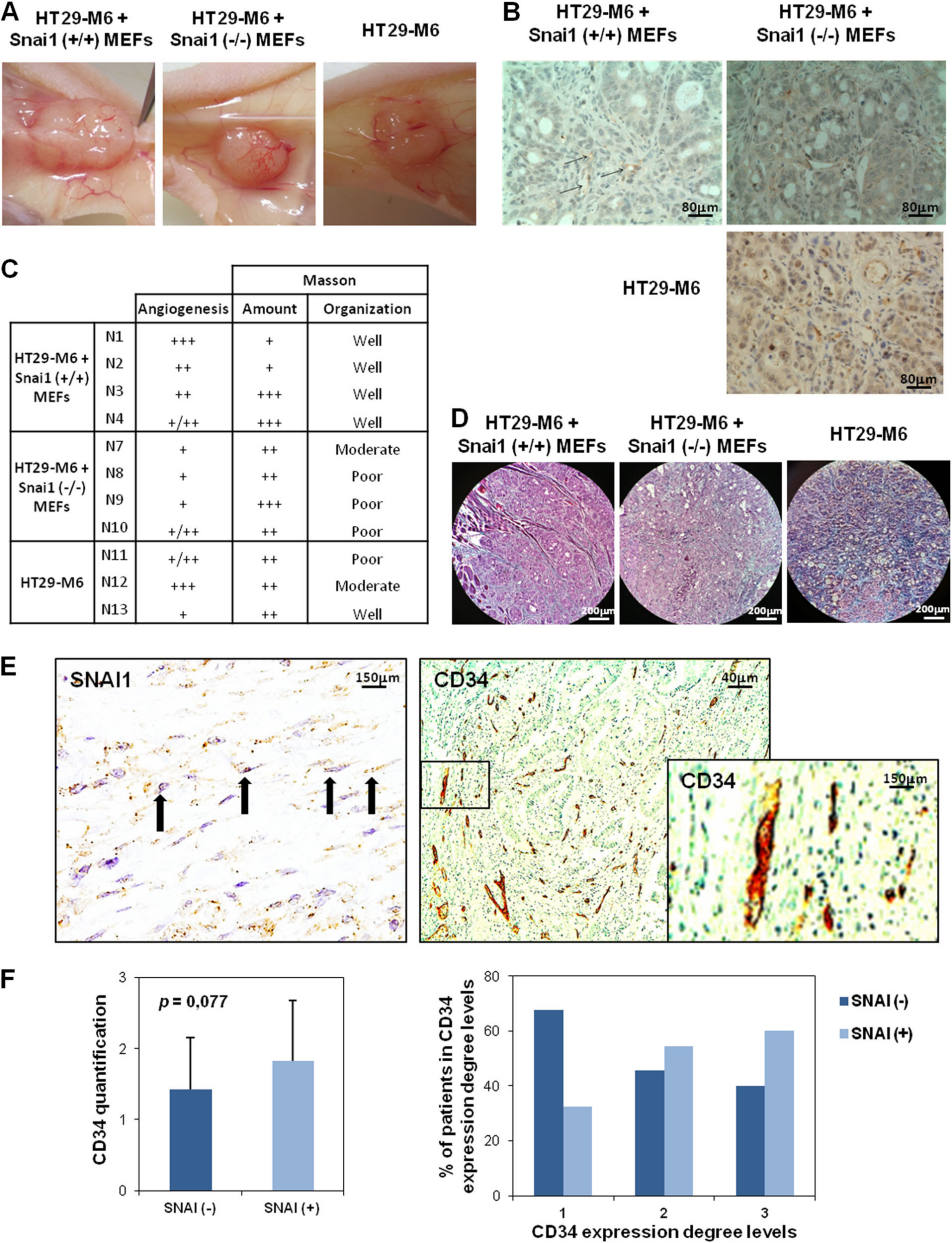
SNAI1 expression stimulates angiogenesis in xenograft tumor models and is associated with angiogenesis markers in human tumor samples. **a**, **b** Macroscopic and microscopic decrease of vascular irrigation in tumors derived from colon cells and Snail1 ko MEFs. **c** PCAM-1 and Masson staining measurement in xenograft tumors. **d** Illustration of Masson staining in Snail1 wt or ko MEFs + tumor colon cell-derived xenograft tumors. **e** Representative images of a patient with SNAI1 expression and high degree of vasculature. **f** Direct association between SNAI1 expression and the degree of vasculature in human colon tumor samples

A remarkable decrease in vascular irrigation was macroscopically observed when we compared tumors generated by co-injection of tumor cells and Snail1 KO MEFs vs. wt MEFs (Fig. 6a). Staining of vascular vessels with PECAM-1 antibody supported the macroscopically observed data (Fig. 6b, c).

Moreover, Masson staining was performed to analyze Collagen I organization in the tumor mice models. Although no differences were observed in the total Collagen I, the organization of this protein depended on Snail1 expression in MEFs. Thus, those tumors derived from epithelial cells and Snail1 KO MEFs exhibited a lower organization than tumors derived formed by HT-29M6 plus SNAI1 wt MEFs (Fig. 6c, d).

Tumors formed by HT29-M6 human colon tumor cells without co-injection of fibroblasts, showed vascular vessel and Collagen I organization between those observed in tumor with co-injection of Snail1 KO or wt MEFs (Fig. 6c), suggesting that Snail1 KO MEFs prevents activation of resident fibroblasts.

Thus, this analysis demonstrated that the Snail1 status of co-injected fibroblasts determined angiogenesis behavior and ECM Collagen I organization of endogenous endothelial cells in xenograft colon cancer models.

### Snail1 expression in fibroblasts is associated with endothelial cell markers in human colon tumor samples

Finally, the expression of Snail1, CD34 and CD31 as angiogenic marker, was analyzed by IHC in a series of 53 colon cancer patients. Snail11 expression in tumor stroma was categorized as presence or absence of expression, given the low number of expressing cells. However, the expression of CD34 and CD31 was measured at three levels, in which “Grade 1” corresponded to those samples with lower staining and “Grade 3” to those with highest expression.

As expected, CD34 and CD31 showed a statistical association (Sup. Fig. 10A). The analysis showed a direct association between SNAI1 presence and CD34 but not with CD31. Thus, SNAI1-positive tumors showed a 1.27 rate of vasculature markers expression, compared with negative SNAI1 samples. Moreover, the percentage of patients with “Grade 3” of CD34, and thus more vasculature, was higher among SNAI1-positive tumors; on the contrary, those tumors without SNAI1 expression presented the greatest percentage of patients with low angiogenesis or CD34 expression (patients with “Grade 1”) (Fig. 6e, f and Sup. Fig. 10B).

## Discussion

In this study, we established for the first time a role for ECM fiber alignment in tumor angiogenesis of colorectal cancer patients *via* the PDGF/FAK/Snail1 pathway. Thus, 3D-ECMs derived from PDGF-stimulated fibroblasts showed increased fiber deposition and alignment organization. Endothelial cell activation and tubulogenesis were observed when these cell were seeded on 3D-ECMs. Interestingly, when we inhibited the FAK/Snail1 expression in fibroblasts, the activation of endothelial cells was blocked by a mechanism involving ECM fiber disorganization (Sup. Fig. 11). The in vivo significance of these findings was supported by results obtained from tumors generated by co-injection of fibroblasts and colon cancer cells; those with Snail1-deficient cells were less likely to induce neovessel formation. Finally, the clinical relevance of these results was proved by the association between Snail1 expression in the stromal compartment and angiogenesis markers in tumor samples from colorectal cancer patients.

Cancer-Associated Fibroblasts (CAFs) are a heterogeneous population of activated fibroblasts present in the tumor microenvironment that promote tumor growth and progression^1–3^. PDGF ligands stimulate tumor stroma recruitment of CAFs and are an important regulator of CAFs^33^. Thus, stromal PDGFR signaling exerts prometastatic effects having a prognostic role in several tumor types^33^. PDGF stimulates the synthesis of ECM proteins, by fibroblasts, such as Hyaluronan^34^, Fibronectin ED-A and Collagen I^35^. ECM dynamics involve changes in the amount, composition or topography of the fibers, which may result in disorganization and deregulation of its essential properties and could lead to abnormal behavior of cells in tumor tissues^19,36^. In line with these data, we demonstrated that BJ-hTERT fibroblasts increase matrix deposition and alignment upon PDGF treatment, which results in an anisotropic organization of both Collagen I and Fibronectin ECM fibers. Supporting these data, and in line with previously described data in which p65 subunit of NF-κB participate in the activation of fibronectin transcription, an increase of p65 phosphorilation was observed in BJ-hTERT fibroblasts under PDGF stimulation. Moreover, data derived by gene expression profile of nonstimulated and PDGF-stimulated BJhTERT fibroblasts supports also these findings, since PDGF stimulation of fibroblasts was involve in the regulation of many genes related with ECM. These changes in ECM properties were also validated when using NFs and CAFs established from normal or tumor samples derived from colorectal cancer patients. As expected from their activated phenotype, CAFs resemble PDGF-treated fibroblast behavior, as they produced parallel fiber alignment and dense matrices unlike NFs. Furthermore, PDGF-treated CAFs showed greater organization of ECM fibers than non-treated CAFs do.

During tumor growth, new blood vessel formation is crucial to face the increasing demand for nutrient, oxygen and waste exchange^37^. The organization and composition of the ECM control endothelial cell activities, such as cell survival and proliferation, vessel lumen formation and tubulogenesis, and provide tracks to guide endothelial cell migration and branching^37^. Similarly β1 integrin subunits is involved with establishment and stiffness of collagen fibers to form ECM scaffold. Moreover, endothelial cell adhesion to collagen and fibronectin are mediated by β1 and β3 integrin subunits which play a pivotal role during tumor angiogenesis and are highly expressed on activated endothelial cells and new-born^38,39^. Despite the above-mentioned findings, the cross-talk mediated by CAFs and ECM fiber organization and its association with the angiogenesis process has not yet been fully studied. In this study, we found that PDGF-activated fibroblasts induced ECM deposition with anisotropic organization of fibers and that these fibers’ alignment favored endothelial cell attachment to ECM and activation, which results in increased tubulogenesis. Accordingly, the tubulogenesis process was not found in HUVECs on matrices from PDGF-stimulated fibroblasts treated with LOX inhibitor. Consistent with our data, it has been described that Collagen I is a potent pro-vascular tube morphogenesis in 3D matrices, increasing tissue stiffness and remodeling ECM to provide a network of tracks that support migration of cells^27,37^. Accordingly, our data showed that PDGF-treated fibroblasts embedded in Collagen I gels generated a stiff ECM, as demonstrated by its increased contraction capacity. However, Western Blot found no changes of PDGF-stimulated fibroblasts or LOX-treated fibroblasts in LOX protein, suggesting that other proteins may be regulating the process of fiber alignment in our model.

Growth factors, such as PDGF, or phosphorylate receptor tyrosine kinases (RTKs), result in activation of three key downstream pathways commonly mutated in tumors: the small GTPase Ras/ERK, the Phosphatidylinositol 3′-Kinase/Akt and the Focal Adhesion Kinase (FAK)^40^. In our study, the Akt inhibitor completely reverted Collagen I deposition, while ERK and FAK inhibitors revealed only a partial blockade. However, when analyzing the organization of ECM fibers, FAK was the main protein regulating ECM dynamics mediated by PDGF, and FAK inhibition led to the disorganization of fibroblasts and fibers. In addition, the tubulogenesis process was completely inhibited on fibroblast 3D-ECMs treated with the FAK inhibitor. Accordingly, survival of endothelial cells during angiogenesis is mediated by FAK^41^ and its deregulation is associated with several carcinomas, including colon carcinoma^42^.

Snail1 is a transcriptional factor that initiates the EMT, a common process observed in several tumors^17^. In fibroblasts, PDGF promotes an activated phenotype and induces Snail1 that acts as a main regulator of both gene expression and functional properties in these cells^23,24^. Since Snail1 expression in fibroblasts predicts outcome of colon cancer patients, we recently proposed it as a marker of activated fibroblasts, which lead to the production of soluble pro-migratory molecules, resulting in cancer cell invasion^18,19,21,43,44^. In addition, Snail1 plays an important role in ECM composition by modifying Collagen and Fibronectin deposition and alignment^19,25,28–31^. Inversely, Collagen I receptor DDR2 prevents Snail1 degradation, reinforcing ECM fiber organization and stiffness^30,31^. We also previously described that expression of Snail1, induced by TGFβ in fibroblasts, is required for the activation of RhoA and the acquisition of a myofibroblastic phenotype. As a consequence, scattered Snail1-expressing fibroblasts impose a mechanical microenvironment needed for breast cancer cell migration and invasiveness^19^. However, no links between Snail1 activity and the modulation of angiogenesis through changes in ECM properties have been found. Currently, we showed that PDGF treatment increases Snail1 expression levels and nuclear translocation in fibroblasts and CAFs. Ectopic Snail expression in human fibroblasts increase in a synergic manner the PDGF regulation on the transcription of several ECM proteins. Moreover, Snail1 depletion in fibroblasts resulted in deregulation of fibroblasts and ECM organization together with a decrease in p65 subunit phosphorilation. Those CAFs in which ligand responses were more clearly observed generated a more aligned pattern of ECM fibers together with a higher increase in Snail1 expression. In addition, HUVECs seeded on 3D-ECMs derived from Snail1 KO fibroblasts did not form capillary-like structures and displayed lower levels of endothelial activation and attachment markers. In support of our data, some evidence has shown an association between Snail1 and angiogenesis in mouse models^45,46,47^.

In addition, although both PI3K/Akt and FAK inhibition decreased the expression of the cytoskeletal protein α-SMA, only the FAK inhibitor decrease Snail1 protein. Thus, blocking FAK in fibroblasts revealed a similar pattern of ECM fibers and tubulogenesis in HUVECs as Snail1-depletion fibroblasts and BAPN-treated ECMs. These data suggest that the PDGF, FAK and Snail1 regulate cell morphology, leading to aligned ECM fibers that serve as tracks for the tubulogenesis process. It has also been demonstrated that Snail1 activates FN1 promoter and also controls α-SMA and the formation of stress fibers^19,25^. In a theoretical loop, the resulting aligned Fibronectin fibers work as a template for the assembly of other ECM molecules, such as Collagen, which are cross-linked by LOX. In turn, the DDR2 Collagen receptor is activated, leading to Snail1 stabilization^19,30^.

During the angiogenic process, the activated cell, called the tip cell, invades the ECM. Adjacent cells, referred to as stack cells, proliferate and follow the tip cell, resulting in the formation of a sprout. This process has been associated with collective endothelial cell migration^47^. In this study, we have shown that PDGF-treated Snail1-expressing fibroblasts generated organized 3D-ECMs, increasing proliferation and collective invasion as a cohort of HUVECs that indicated the activation of the sprout process in endothelial cell. However, despite the present study and previous ones, the molecular pathways and the spatiotemporal regulation of angiogenic morphogenesis remain largely unknown.

Xenografted tumors generated by co-injection of Snail1-deficient fibroblasts and colon cancer cells were less able to induce neovessel formation and to organize the Collagen I fibers in the ECM. These data strongly supports the in vivo significance of the present study and suggest that these Snail-expressing fibroblast effects might be mediated by ECM remodeling and angiogenesis regulation. The data observed with HT29-M6 cells without co-injection are also in accordance since in these tumor the stroma was formed by resident fibroblasts of the mice. Our data, showing that tumors formed with HT29-M6 and KO MEFs displayed lower angiogenesis than tumors with only HT-29M6 (but less than with wt MEFs) suggest that KO MEFs are protecting resident fibroblast from activation. A similar results has been obtained in a model of breast tumor where MEFs KO protected from epithelial tumor metastasis^48^.

Finally, the analysis of human tumor samples showed an association between Snail1 expression in the stromal compartment and the angiogenesis marker CD34, but not with CD31. CD34 is expressed in vascular endothelial progenitors while CD31 is expressed in most of endothelial cells^49^. This suggests that Snail1, as CD34, would be a marker of neoangiogenesis, but not of regular vasculature, as CD31. Although these analyses need to be extended with larger patient series, the increase of angiogenesis in those tumors with the highest degrees of Snail1 expression supports evidence for the biological effects of Snail1-expressing fibroblasts on endothelial cell activation.

Experimental studies have brought some controversy about PDGFR-β function in endothelial cells. We did not observe PDGFR-β expression in cultured HUVECs upon PDGF treatment or under co-culture with fibroblasts. Our results are in line with the analysis in knockout mice that do not show evidences for PDGF signaling in endothelial cells^50^. Thus the expression of Snail protein as a mediator between PDGF and endothelial cell activation might be an explanation for the confusion of PDGF functions in endothelial cells.

Thought the inhibition of the PDGF ligand was successful in therapeutic angiogenesis in diverse preclinical models, the expected results failed to occur in clinical trials^26^. According to our results this might be consequence of the different fibroblast infiltration in tumors and the expression of angiogenic mediators. Therefore, the analysis of Snail1 expression in tumor stromal fibroblasts might be used as a potential biomarker to identify sub-groups of patients who might respond better to therapy and thus to improve personal treatments.

## Materials and methods

### Reagents

Primary antibodies used in this work were β-actin (Abcam, ab8226), SNAI1 EC3 (ref^20^.), PY99 (Santa Cruz, sc7020), PDGFR-β (Cell Signaling, 3169), ERK1/2 (Cell Signaling, 9102), p-ERK1/2 (Cell Signaling, 9109), Akt (Cell Signaling, 4691), p-Akt (Cell Signaling, 4060), FAK (Cell Signaling, 3285), p-FAK (Cell Signaling, 3281), VE-Cadherin (Abcam, ab33168), VCAM-1 (Abcam, ab98954), MMP-9 (Abcam, ab38898), Snail (ref^20^.), MT1-MMP (Abcam, ab51074), CD31/PCAM-1 (1:10, SC-506) Fibronectin (DAKO, A0245), Beta 1 integrin (Cell Signaling, 34971 S), Beta 3 integrin (Cell signaling, 13166 S), NF-KB p65 (Cell signaling, 8242 S), Phospho-NF-KBp65 (Cell signaling 3033 S) and Collagen I (Abcam, ab34710). Secondary antibodies used were anti-Mouse or anti-Rabbit IgG Antibody DyLight™ 680 or 800 Conjugate.

Other compounds used were PDGF-BB (Peprotech, 100-14B), BAPN: 3-Aminopropionitrile fumarate salt (Sigma, A3134), FAK inhibitor, PF-573228 (PZ0117, Sigma), ERK inhibitor, U0126-monoethanolate (Sigma, U120) and PI3K/Akt inhibitor LY294002 hydrochloride (Sigma, L9908).

### Tube formation assay on 3-D matrices

For the angiogenesis assay on 3-D fibroblast-derived matrices, HUVECs were maintained in EGM-2 containing 2% FCS. After 8 h of starving in EGM-2 without FCS, 2 × 10^5^ cells/well were seeded in FCS-depleted medium onto 6-well tissue culture plates coated with 3-D fibroblast-derived matrices. Cells were cultured for 16 h and then capillary-like tubes were analyzed.

### Proliferation assay on 3-D matrices

HUVEC cells were seeded at a density of 15,000 cells/well onto 96-well tissue culture plates coated with 3-D fibroblast-derived matrices from Snail1 wt and KO MEFs. After 72 h proliferation was analyzed. For details see Supplementary Information.

### Organotypic co-culture invasion assay on collagen gel

Collagen gels were prepared by mixing 5 volumes of type I Collagen (3 mg/ml Sigma), 2 volumes of DMEM 10% FBS, 1 volume of Reconstitution Buffer (50 mM NaOH, 260 mM NaHCO3, 200 mM HEPES) and 1 volume of FBS with fibroblasts (resuspended at a density of 5 × 10^5^ cells/ml). 1.8 ml of the gel mixture was placed in each transwell-insert of a 6-well plate and allowed to polymerize at 37 °C for 1 h. After the gel solidified, the bottom chamber was filled with 2.5 ml of 3D co-culture medium. Then, HUVECs were resuspended in 3D-medium (1:1 of DMEM 10% FBS and EGM-2 2% FBS) at a concentration of 2 × 10^5^ cells/ml, added to the gel and incubated at 37 °C. Every 2–3 days, medium from both well and insert was replaced by 3D co-culture medium containing PDGF treatment (10 ng/ml). After 6 days, the cultures were “lifted” to the air–liquid interface by adding 1 ml media to the bottom chamber only, allowing the airway endothelial cells seeded on the apical chamber to be exposed to air. The cells were allowed to grow for an additional 5 days and were fixed in formalin solution overnight at room temperature and embedded in paraffin. Vertical sections (4 μm) with hematoxylin and eosin (H&E) were stained.

Other methods, including “Culture cell lines2, “Stromal Fibroblast-Derived 3-D Matrix Production”, “Western Blotting and Immunofluorescence details”, “Generation of Snail1 modified cell lines and PCR array”, Proliferation assay”, “In vivo xenograft tumor model”, “Immunohistochemistry and Masson’s procedures” and “Statistical analysis” are described in the Supplmentary Information.

## Electronic supplementary material

Supplemental information

Supplemental Figure 1

Supplemental Figure 2

Supplemental Figure 3

Supplemental Figure 4

Supplemental Figure 5

Supplemental Figure 6

Supplemental Figure 7

Supplemental Figure 8

Supplemental Figure 9

Supplemental Figure 10

Supplemental Figure 11

Supplemental Table 1

Supplemental Table 2
