# Supplementary material for: Endothelial cell activation on 3D-matrices derived from PDGF-BB-stimulated fibroblasts is mediated by Snail1
Source: Oncogenesis. 2018 Sep 24;7(9):76. doi: 10.1038/s41389-018-0085-z (PMC6155204; doi:10.1038/s41389-018-0085-z)
Supplement: Supplementary file 1 — Supplemental information [file 41389_2018_85_MOESM1_ESM.docx]

**SUPPLEMENTARY METHODS**

**Culture cell lines**

BJ-hTERT fibroblasts, MEFs and HT29-M6 were cultured in Dulbecco’s modified Eagle medium (DMEM, Lonza), supplemented with 10% Fetal Bovine Serum (FBS), 2 mM L-glutamine, penicillin (100 U/ml) and streptomycin (100 ng/ml) at 37ºC in a 5% CO2 humidified atmosphere. HUVECs were cultured in same conditions with EBM-2 (lonza) and 1.BR3.G immortalized human dermal fibroblasts with EMEM (Lonza).

BJhTERT and HT29-M6 cells were obtained from commercial provider (American Type Culture Collection). 1.BR3G fibroblasts cell line were obtained from Cancer Cell Line Repository from IMIM, Barcelona, Spain (http://www.blc.imim.es/). Certificates of the “Cell Line STR Profile” from HT29 colon tumor cells, and from BJhTERT and 1BR3G human fibroblasts were assessed. HUVECs were obtained from commercial provider (American Type Culture Collection), and as primary cells, there are not STR profile available to check them. Passage numbers were kept at a minimum.

Snail1 wt or KO Mouse Embrionic Fibroblasts are derived from a mouse bearing a Snail1-floxed form of this gene (Stanisavljevic et al, Cancer Research 2015). MEFs were used at low passages and without experimentally induced or spontaneous immortalization.

Established human primary Cancer Associated Fibroblasts and Normal Fibroblasts from tumor and normal colon mucosa are short-term cultures of human tumors.

**Human samples**

Fresh tissue from 8 patients operated for colorectal primary tumors at the Puerta de Hierro-Majadahonda University Hospital was used for the propagation of primary CAFs and NFs. CAF establishment and growth details were performed as described in (Herrera et al, Bioprotocol 2016: <http://www.bio-protocol.org/e1773>). The study of paraffin embedded tissues section is based in a series of 60 patients who had surgery for colon cancer between January 2000 and January 2012. Informed written consent was obtained from all participants after an explanation of the nature of the study, as approved by the Research Ethics Board of Puerta de Hierro Majadahonda University Hospital.

**Stromal Fibroblast-Derived 3-D Matrix Production**

BJ-hTERT or MEFs were cultured in Dulbecco’s modified Eagle medium (DMEM) containing 10% FCS media and penicillin/streptomycin antibiotics under standard conditions. Tissue culture plates were first pretreated with 0.2% gelatin solution (Sigma-Aldrich, St. Louis, MO, USA) for 1 h at 37°C, followed by 1% glutaraldehyde (Sigma) and 1 M ethanolamine (Sigma) for 30 min separately at room temperature. Fibroblasts were seeded at a concentration of 2×10^5^ cells onto 6-well pretreated plates in DMEM medium supplemented with 10% fetal bovine serum, 100 U/mL penicillin, 100 μg/mL streptomycin and 0.25 μg/mL fungizone. Confluent fibroblast cultures were treated with fresh maintenance media supplemented with 50 μg/ml of cell culture-tested ascorbic acid (Sigma-Aldrich), and PDGF-BB 20 ng/ml (Peprotech) when necessary, every other day for 6 days. Fibroblasts remove from matrices was performed by an incubation with an alkaline buffer (0,5% (v/v) Triton X-100 and 20nmol/L NH_4_OH) during 10 minutes at 37ºC. The resulting extracted 3-D matrices were blocked with heat-denatured 2% BSA and stored at 4°C in PBS containing 100 U/ml penicillin and 100 μg/ml streptomycin until needed. After 3-D matrices isolation, HUVECs were seeded to accomplish the sprouting assay.

**Western Blotting and Immunofluorescence details**

For Western Blotting, the medium of the cells was removed carefully and two washes of ice-cold PBS were performed. Then, cells were lysed in RIPA buffer supplemented with Halt protease inhibitor cocktail (Thermo Fisher Scientific, Waltham, MA, USA) for 15 minutes on ice. Protein extracts were clarified by centrifugation at 14,000 rpm for 15 min, boiled for 4 min and resolved by electrophoresis in a 10% gradient gel. The proteins were then transferred to nitrocellulose membranes with the iBlot Dry Blotting System (Thermo Fisher Scientific) and following the manufacturer’s instructions. Membranes were incubated for 1 hour in Odyssey Blocking Buffer (LI-COR Biosciences; Pittsburgh, NE). After blocking, membranes were incubated overnight at 4°C with assorted primary antibodies in TBS containing 0.05% Tween-20 (TBST) and Blocking Buffer, followed by three consecutive washes in TBST. Secondary antibodies used were Rabbit or mouse IgG (IRDye700 and IRDye800, Rockland Immunoc.). The protein bands were visualized and quantified by densitometry using (Odyssey Infrared Imaging System (Li-COR Bioscience).

For Snail1 immunofluorescence, cells were fixed with a mixture of 4% paraformaldehyde and 100% methanol (1:1) at -20°C for 10 min, permeabilized with 1% sodium dodecyl sulfate, denatured with 6 M urea and 0.1% glycine, pH 3.5, for 1 h at 4°C, blocked with 3% goat serum, and incubated with the monoclonal antibody SNAI1 EC3 (Franci et al, Oncogene 2006) overnight followed by detection with an Alexa Fluor 488–labeled anti–mouse secondary antibody (Invitrogen). Confocal images of cells were acquired on a confocal microscope (TCS SP5, Leica Microsystems) and a digital camera (DS-L2, Nikon).

For immunofluorescence of 3D-matrices, MEFs were allowed to produce their own matrix (three-dimensional matrix assembly). Fibroblasts remove from matrices was performed by an incubation with an alkaline buffer (0,5% (v/v) Triton X-100 and 20nmol/L NH_4_OH) during 10 minutes at 37ºC. The three-dimensional matrices were fixed with 4% paraformaldehyde, blocked with Blocking Buffer and stained with primary and secondary antibodies overnight at 4ºC. A laser confocal microscope (Leica) was used to image three-dimensional samples and the Odyssey Infrared Imaging System (Li-COR Biosciences) measured fibronectin and Collagen I expression on 3D-matrices.

**Generation of Snail1 modified cell lines and PCR array:**

1.BR3.G immortalized human dermal fibroblasts were transfected with an expression vector for mouse Snail1 (pcDNA3-Snail1-HA) (Batlle E et al, Nat Cell Biol 2000) or with the empty vector (pcDNA3) using Lipofectamine 2000 Reagent (Invitrogen, Carlsbad, CA) following manufacturer’s instructions and then treated with 20 ng/ml PDGF-BB for 24 h.

The PCR array used was “TaqMan™ Array Human Extracellular Matrix & Adhesion Molecules” (Thermo Fisher, 4414133) and the analysis was performed following manufacturer’s instructions in a 7500 Fast & 7500 Real-Time PCR System (Applied Biosystems technology).

**Proliferation assay**

After 8 hours of starving, HUVEC cells were seeded at a density of 15,000 cells/well, in EGM-2 (lonza) with 2% FCS, onto 96-well tissue culture plates coated with 3-D fibroblast-derived matrices from Snail1 wt and KO MEFs. After HUVECs were cultured for 72 h, the medium was replaced by medium containing CellTracker™ Green CMFDA fluorescent dye (Invitrogen, Carlsbad, CA), following the manufacturer's protocol. After 30 min, the medium with green dye was removed and 2 washes were performed before measurement of fluorescence at 535 nm after excitation at 485 nm, using a fluorescence reader (Tecan Infinite F200 Pro; Tecan Group Ltd., Morrisville, NC).

***In vivo* xenograft tumor model**

One million HT-29 M6 cells alone or together with 10^5^ MEFs were subcutaneously injected into the flank of 8-week-old athymic female nude mice (N=6 for each condition). Number of animals were calculated regarding probability of tumor engagement and implementing the principles of replacement, reduction and refinement (‘three Rs’) (EU Directive 2010/63/EU). No randomization even was applicable in the study since the equality of animals. Tumor growth was followed every day after the first week. Animals in which tumor were not developed after one month were excluded from the study. The evaluation of the experiment was done by two investigators blind to the experiment condictions. The study was approved by the Animal Research Ethical Committee of the PRBB.

**Immunohistochemistry and Masson’s procedures**

Sections fixed in 4% buffered paraformaldehyde, embedded in paraffin and sectioned at 3 µm thickness, were stained with Masson’s trichrome stain in line with the standard procedure for observing differences in the development of abnormal Collagen in tissue. For the immunohistochemical analyses, sections were deparaffined and treated with peroxidase blocking solution (Dako) to inhibit endogenous peroxidase activity, blocked in 1% BSA-PBS, followed by incubation with primary and secondary antibodies. The results of these analysis were analyzed by two blind to the experiment conditions. For IHC, percentage of positive cells/ field in 10 fields (200X) was quantified. Results are expressed as: (+) up to 30% of positive cells, (++) from 30-60% of positive cells, (+++) from 60-100% of positive cells. To assess Masson amount and organization the evaluators checked all samples to categorize results for amount of staining: “+”, “++”, “+++”; and levels of organization: “Poor”, “Moderate” and “Well”.

**Statistical analysis**

All cell line experimental data were contrasted using the Student's T-test. All T-tests were performed after evaluation of equality of variance with Levene’s test. Two-tailed P-values ≤0.05 were taken as giving statistical significance.

Directionally histograms of binary pictures of fibroblasts cells and protein fibers were analyzed with Image J software.

*SNAI1*, expression levels in human samples were evaluated as negative or positive since the low expression of the protein. The statistical association among SNAI1, CD31 and CD34 were studied by Kruskall-Wallis Chi-square tests.

All statistical results are derived from 2-4 independent experiments, each performed in duplicate.

Statistical analysis used the SPSS statistical package, version 14.0

**SUPPLEMENTARY FIGURE AN TABLE LEGENDS**

**Supplementary Figure 1.** Increase of p65 phosphorilation in BJ-hTERT fibroblasts after PDGFBB stimulation.

**Supplementary Figure 2.** PDGF do not affect fibroblast number in the conditions used for ECM generation.

**Supplementary Figure 3.** Lack of PDGF-β receptor expression in HUVECs, independently of culture conditions.

**Supplementary Figure 4.** (A) There was no effect of LOX inhibitors in fibroblast growth or organization. B, C) Fibronectin organization (B) and deposition (C) were not affected by LOX inhibitor. D) The LOX inhibitor did not affect LOX protein expression. Directionality histograms (below pictures) were calculated with the Image J software and represent the frequency of distribution of cell angles (A) or protein fibers (B) (centered on the 0 ° angle).

**Supplementary Figure 5.** Inhibition of phosphor-ERK (10 min), -AKT (10 min) and –FAK (24h) by specific inhibitors.

**Supplementary Figure 6.** (A) There was no effect of ERK and AKT inhibitors in fibroblast growth or organization. However, the FAK inhibitor showed a blockage in fibroblast organization. B, C) Collagen I and Fibronectin were not affected by ERK or AKT inhibitors. Directionality histograms (below pictures) were calculated with the Image J software and represent the frequency of distribution of cell angles (A) or protein fibers (B) (centered on the 0 ° angle).

**Supplementary Figure 7. SNAI1 expression in PDGF-treated fibroblasts.** A) Protein SNAI1 expression increase in PDGF-stimulated fibroblasts. At the right the average ± SD of 3 experiments is shown. B) SNAI1 mRNA was determined by qRT-PCR. The figures shows e average ± SD of 3 experiments. C) SNAI1 nuclear translocation in PDGF-treated fibroblasts. C, D) Significant inhibition of SNAI1 nuclear translocation (C) and protein expression (D) in fibroblasts under PDGF and FAK inhibitor treatment.

**Supplementary Figure 8.** Decrease of p65 phosphorilation in SNAI1 KO MEFs regarding wt MEFs.

**Supplementary Figure 9.** Increased of Collagen I and Fibronectin organization in CAFs regarding NFs determined by immunofluorescence in primary human cultures.

**Supplementary Figure 10. CD31 and SNAI1 expression in colon human tumor samples.** A) Association of CD34 and CD31 endothelial markers. B) No association between SNAI1 expression and CD31 endothelial marker in human colon tumor samples.

**Supplemental Figure 11. Endothelial cell activation on 3D-matrices derived from PDGF-BB-stimulated fibroblasts is mediated by Snail1.** A) 3D-ECMs derived from non-stimulated fibroblasts show fiber disorganization without endothelial cell activation nor proliferation. B) PDGF stimulation increase SNAI1 expresion in fibroblasts. SNAI1 regulates activation of NFκB and deposition of ECM proteins and also their alignment. FAK activity is required for Snail1 basal expression. Aligned crosslinked fibers in ECM induces activation and proliferation of endothelial cells. C) Depletion of SNAI1 in fibroblasts prevent the organization of ECM matrix and inhibits endothelial cell activation and proliferation.

**Supplementary table 1.** Gene Set Enrichment Analysis (GSEA) of de-regulated genes in PDGF-BB-stimulated BJ-HTERT fibroblasts.

**Supplementary table 2.** ECM gene expression array of 1.BR3G SNAI1 trasnfected fibroblasts under PDGF-BB stimulation. Data normalized to UBC, HBMS and SDHA housekeepings. Synergic effect of SNAI1 and PDGF-BB are observed in several genes boiled marked.
