# Supplementary figures and images for: Endothelial cell activation on 3D-matrices derived from PDGF-BB-stimulated fibroblasts is mediated by Snail1
Source: Oncogenesis. 2018 Sep 24;7(9):76. doi: 10.1038/s41389-018-0085-z (PMC6155204; doi:10.1038/s41389-018-0085-z)

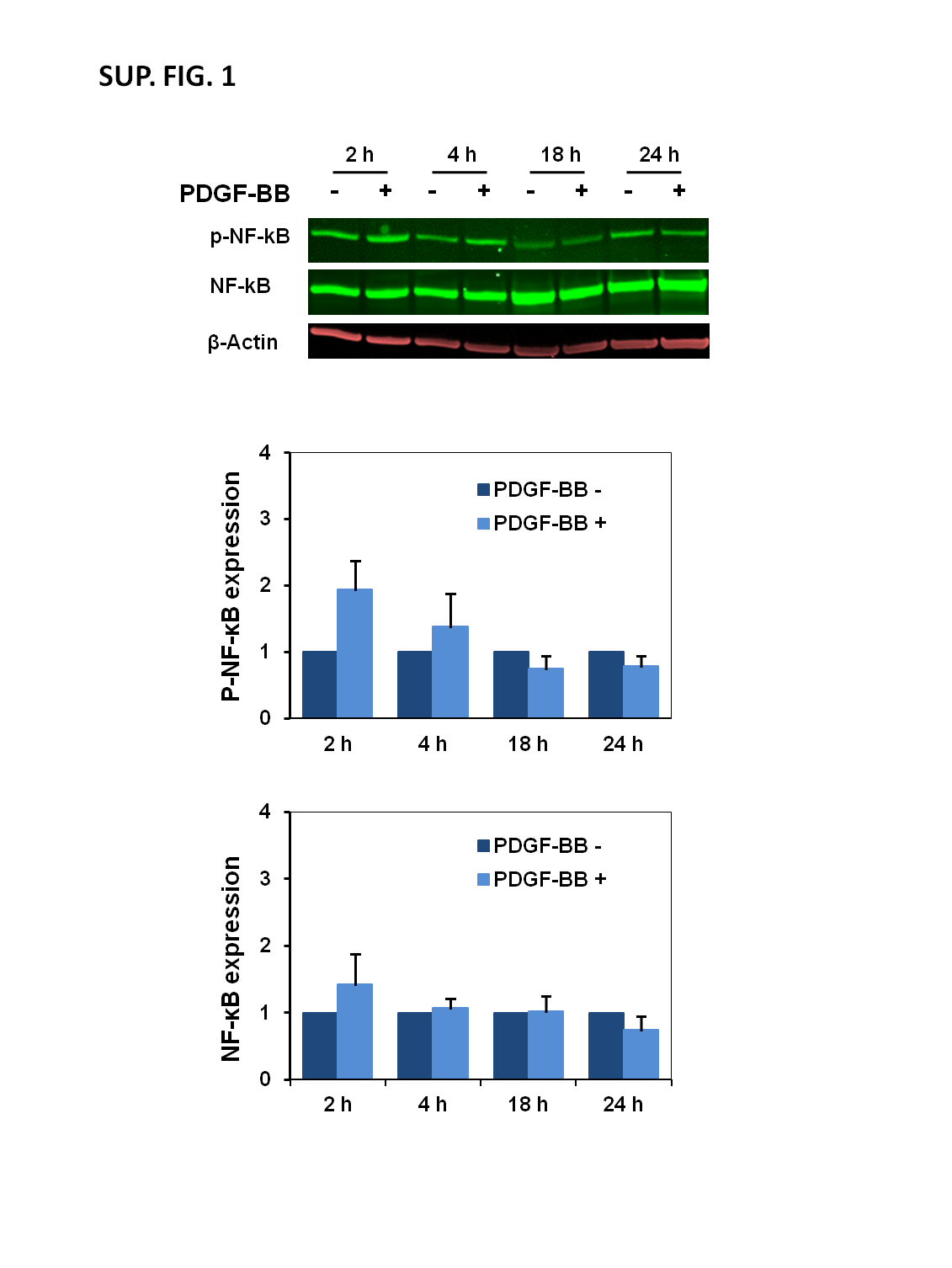

Supplement: Supplementary file 2 — Supplemental Figure 1 [file 41389_2018_85_MOESM2_ESM.tif]

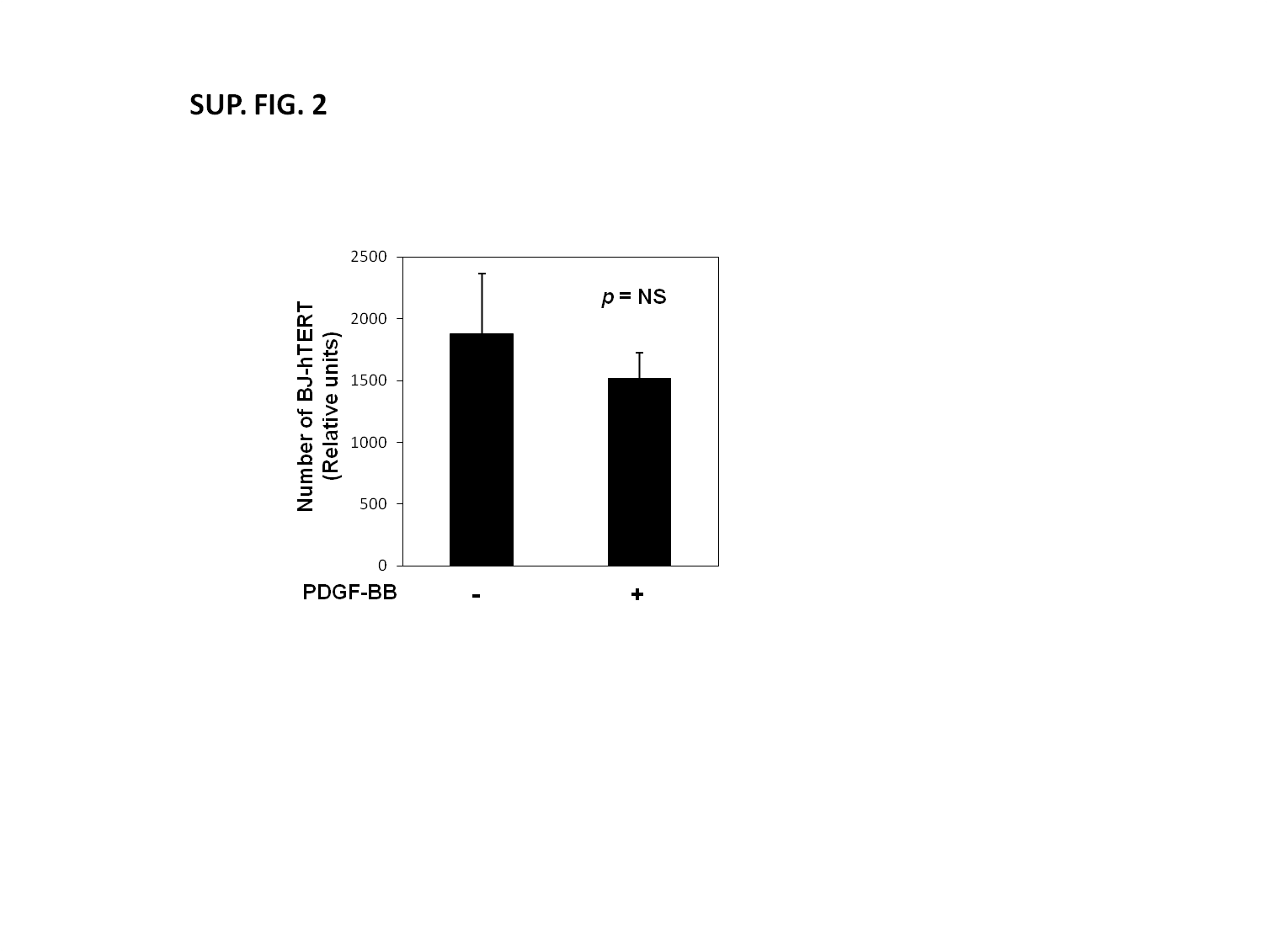

Supplement: Supplementary file 3 — Supplemental Figure 2 [file 41389_2018_85_MOESM3_ESM.tif]

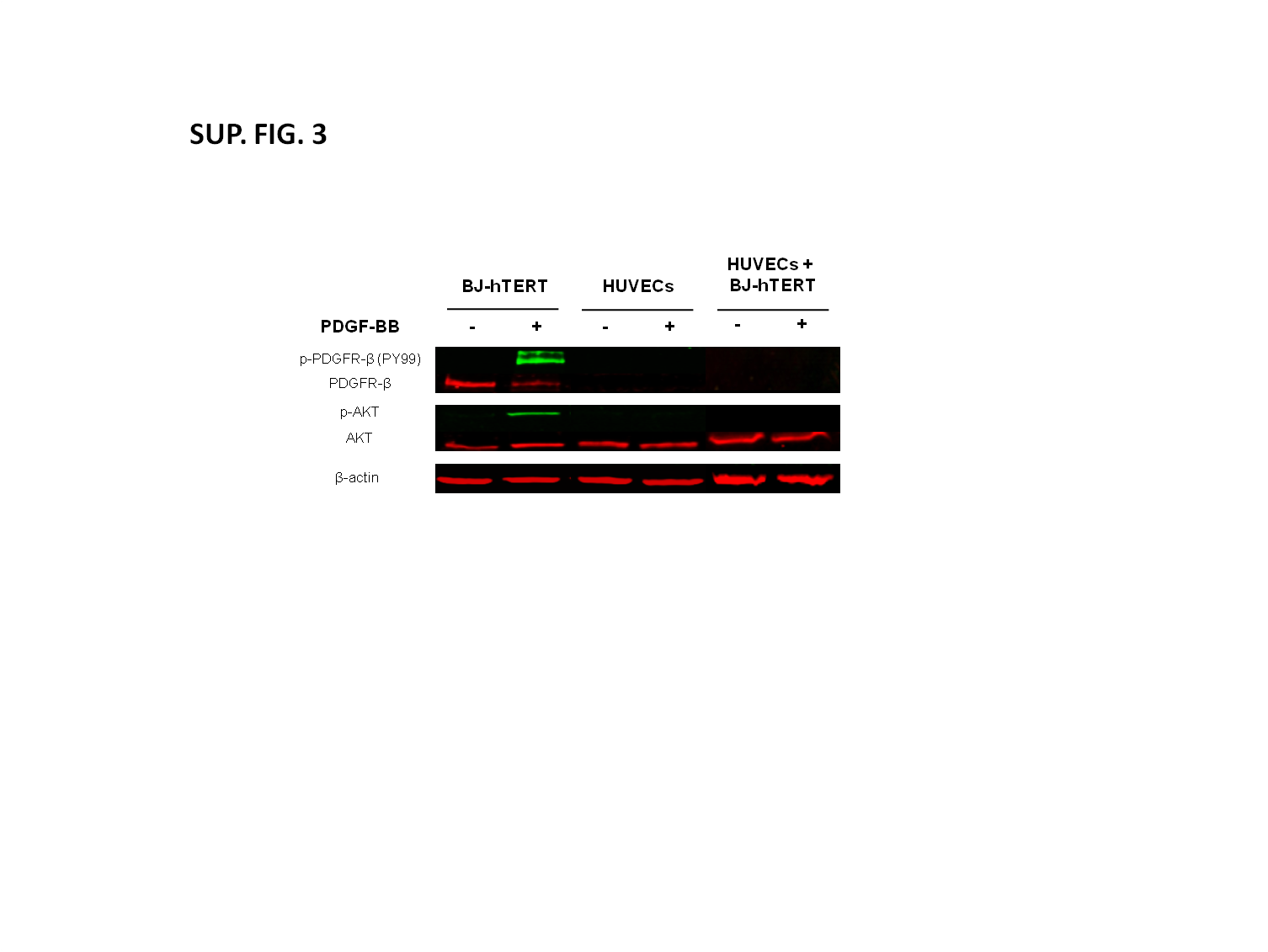

Supplement: Supplementary file 4 — Supplemental Figure 3 [file 41389_2018_85_MOESM4_ESM.tif]

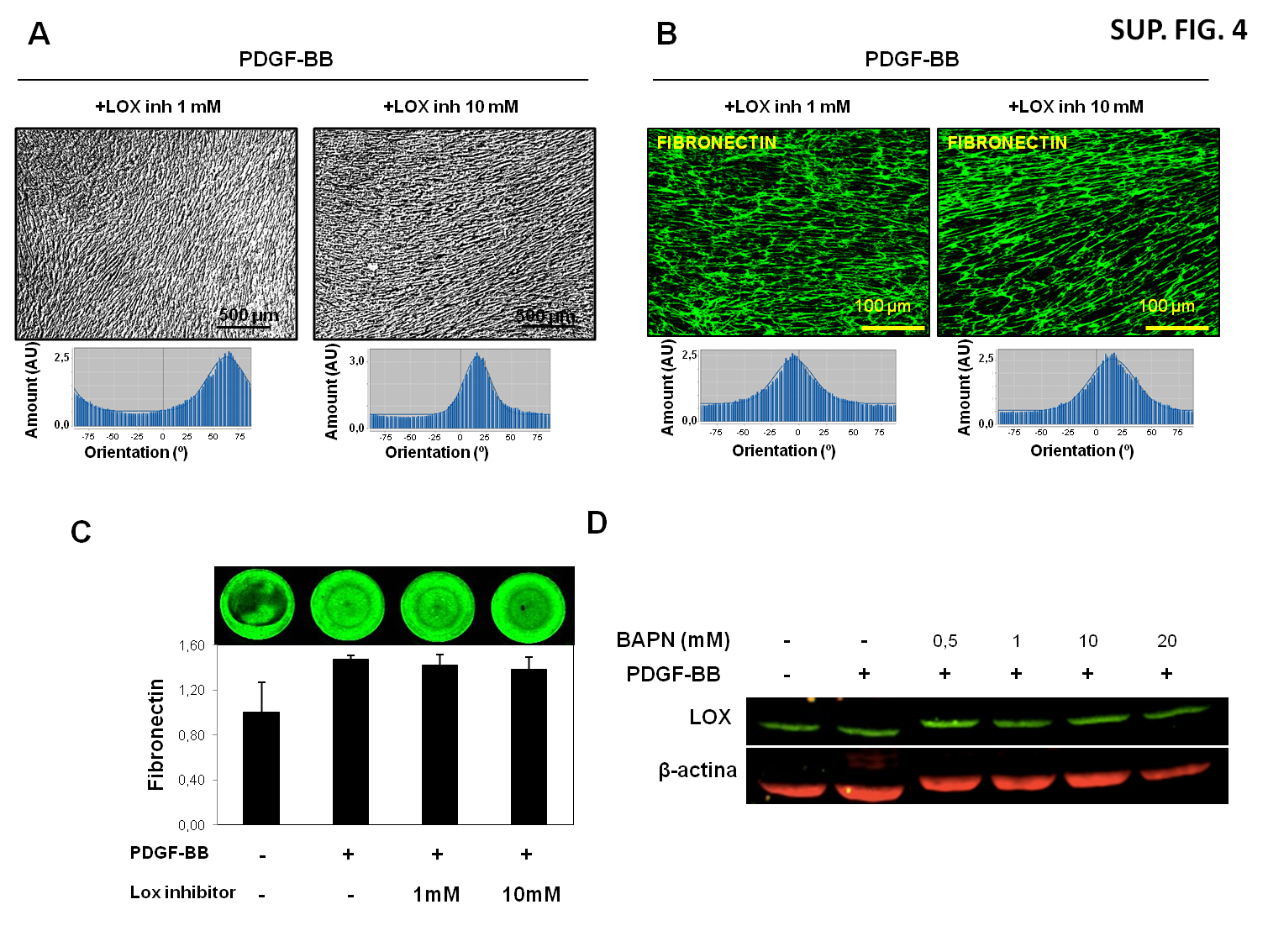

Supplement: Supplementary file 5 — Supplemental Figure 4 [file 41389_2018_85_MOESM5_ESM.tif]

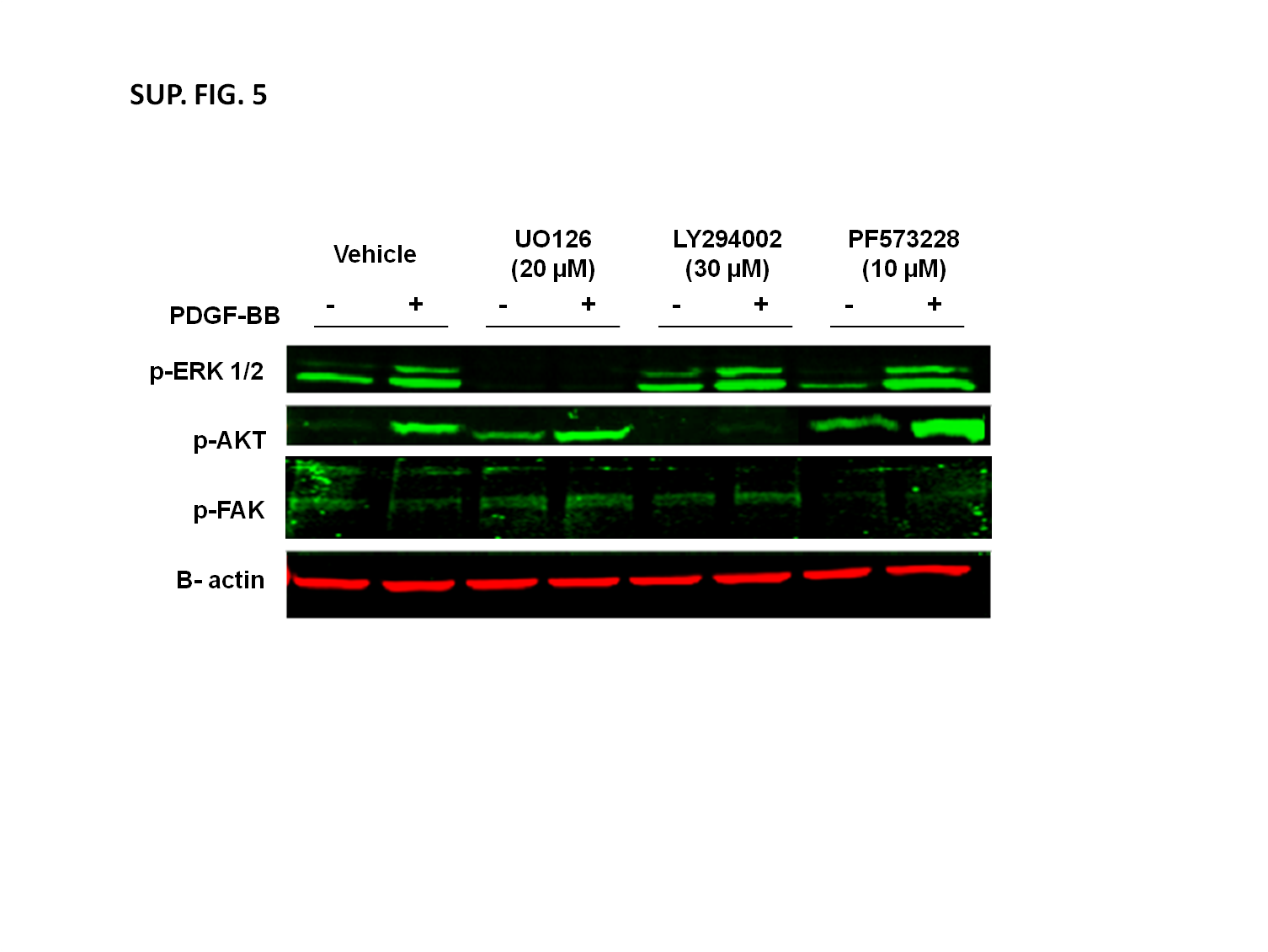

Supplement: Supplementary file 6 — Supplemental Figure 5 [file 41389_2018_85_MOESM6_ESM.tif]

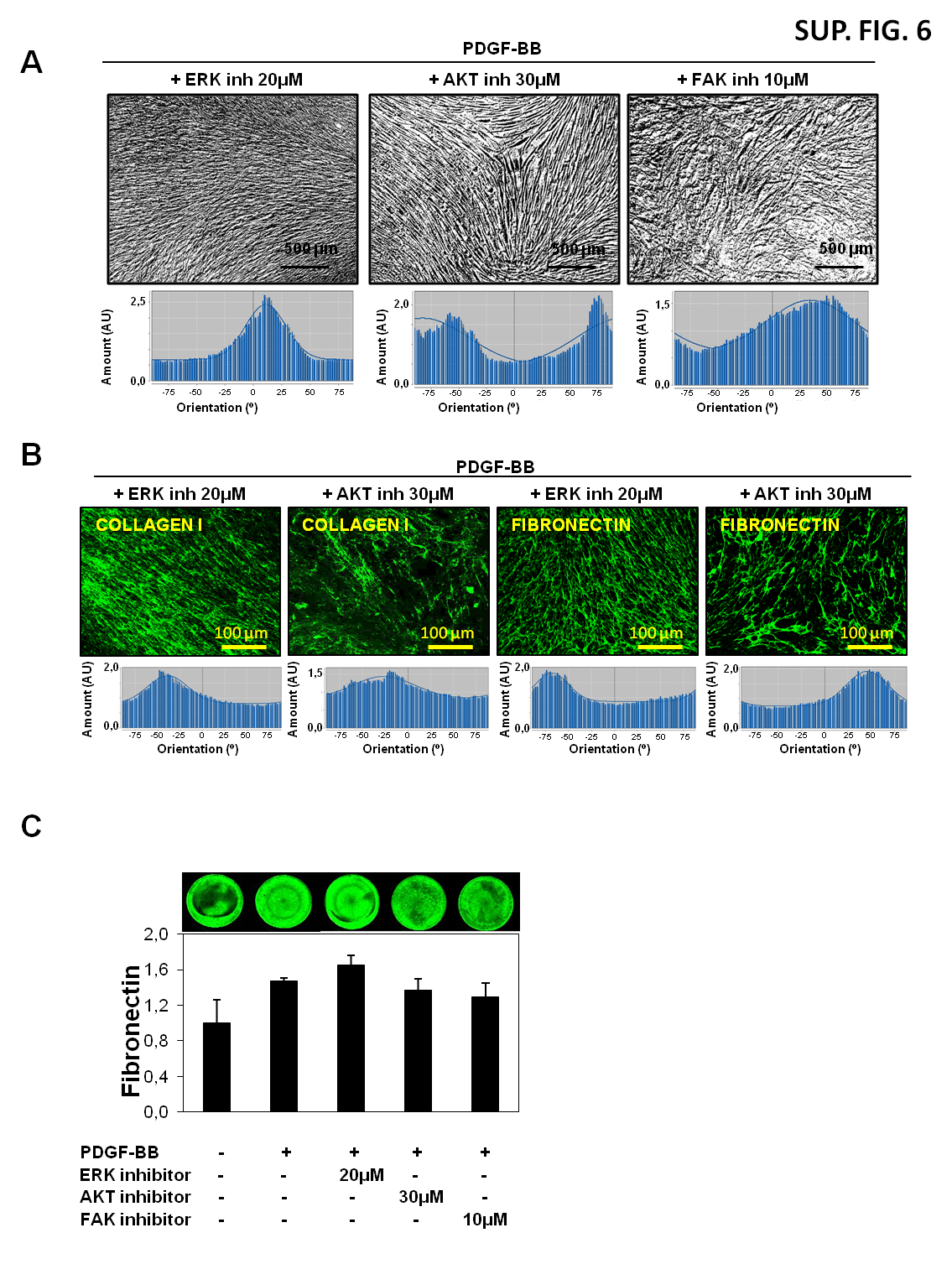

Supplement: Supplementary file 7 — Supplemental Figure 6 [file 41389_2018_85_MOESM7_ESM.tif]

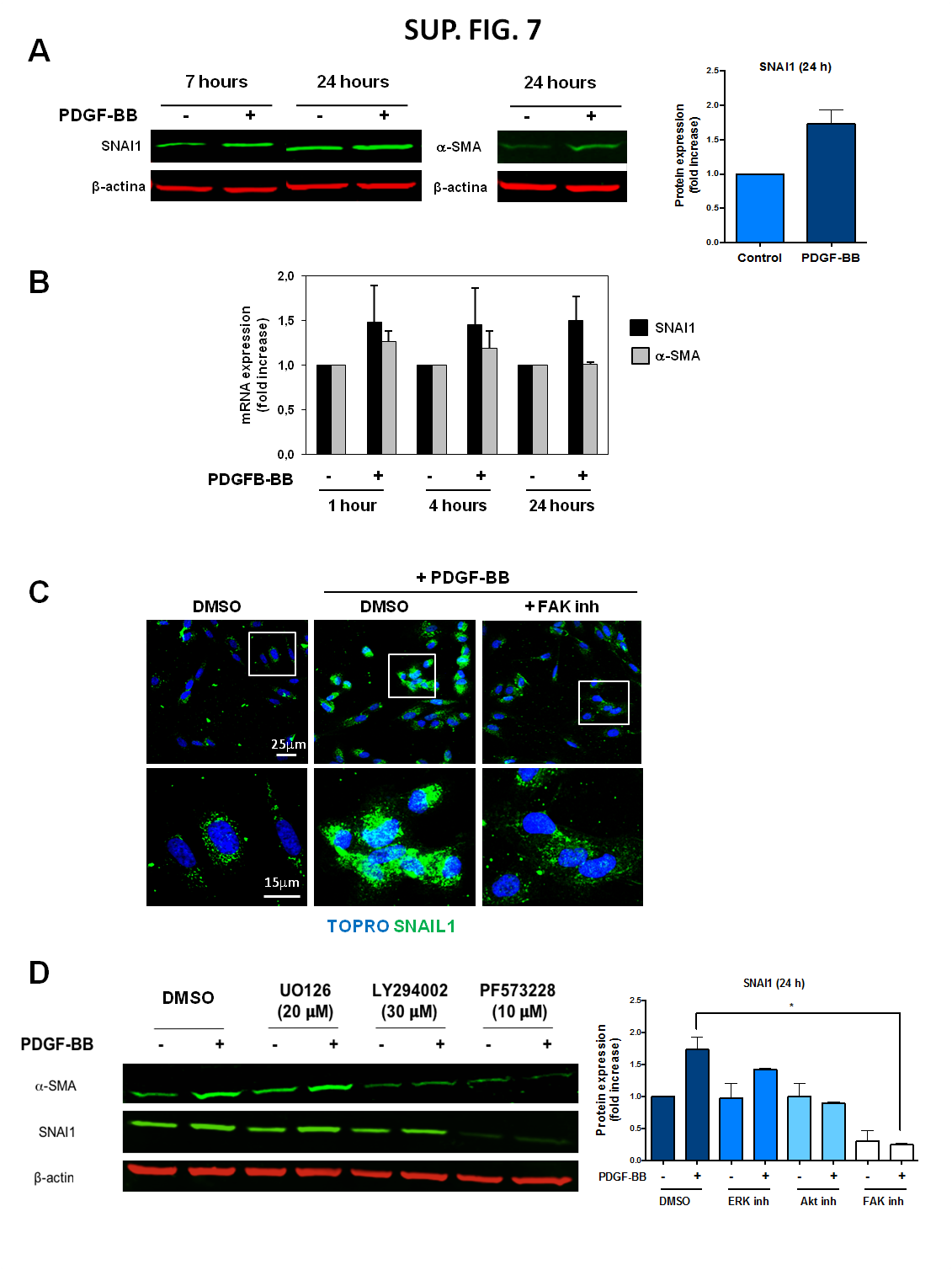

Supplement: Supplementary file 8 — Supplemental Figure 7 [file 41389_2018_85_MOESM8_ESM.tif]

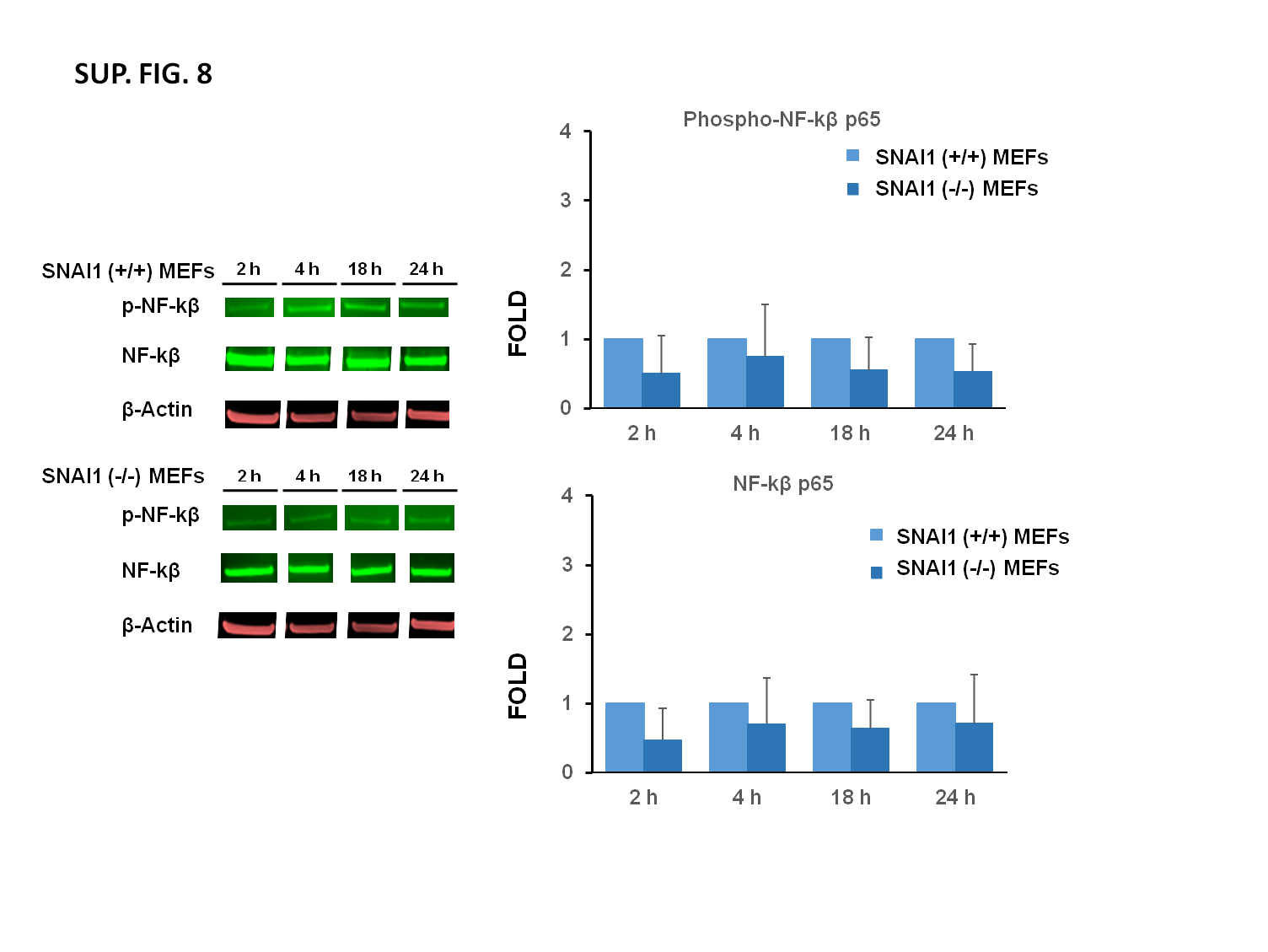

Supplement: Supplementary file 9 — Supplemental Figure 8 [file 41389_2018_85_MOESM9_ESM.tif]

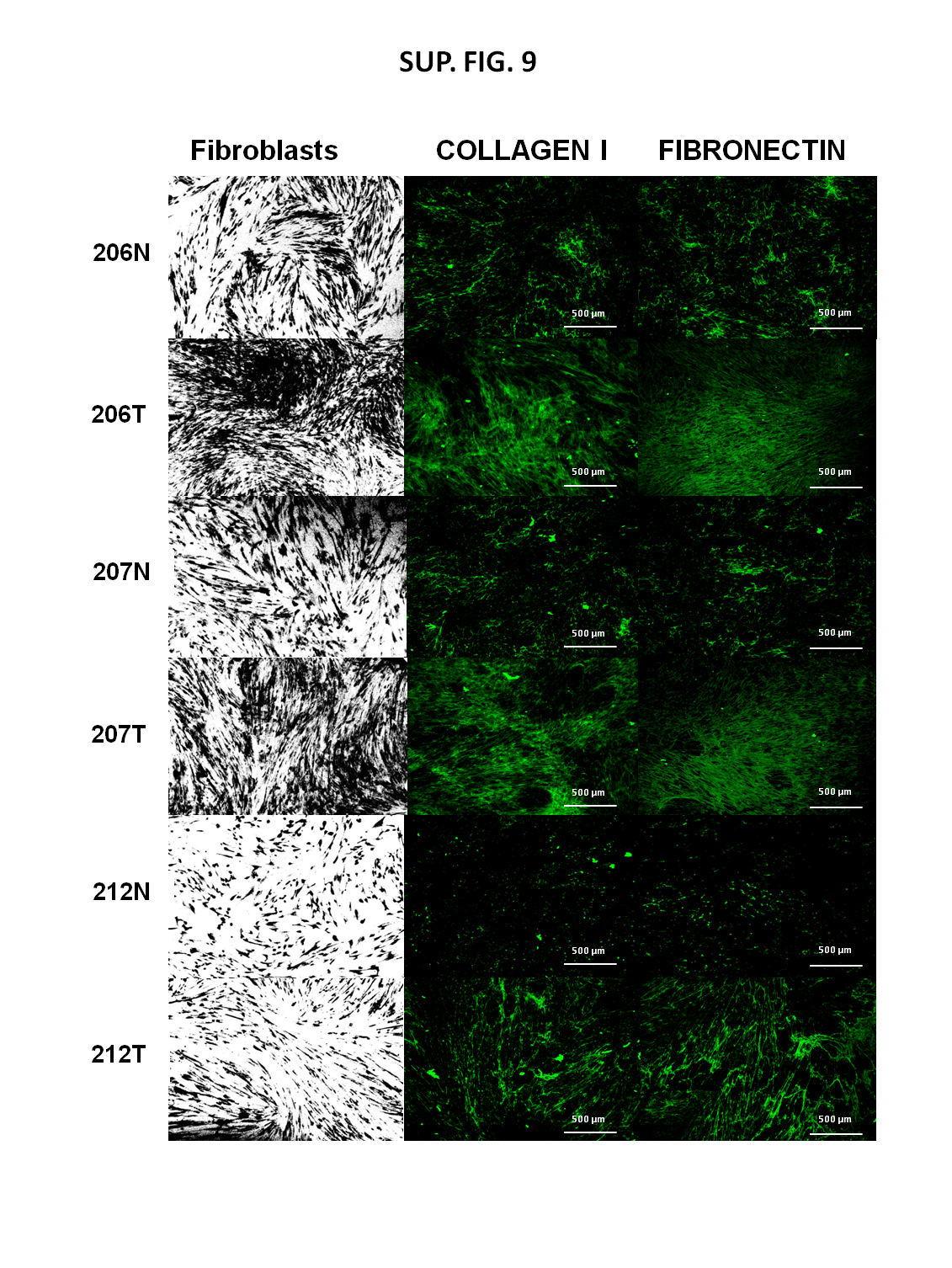

Supplement: Supplementary file 10 — Supplemental Figure 9 [file 41389_2018_85_MOESM10_ESM.tif]

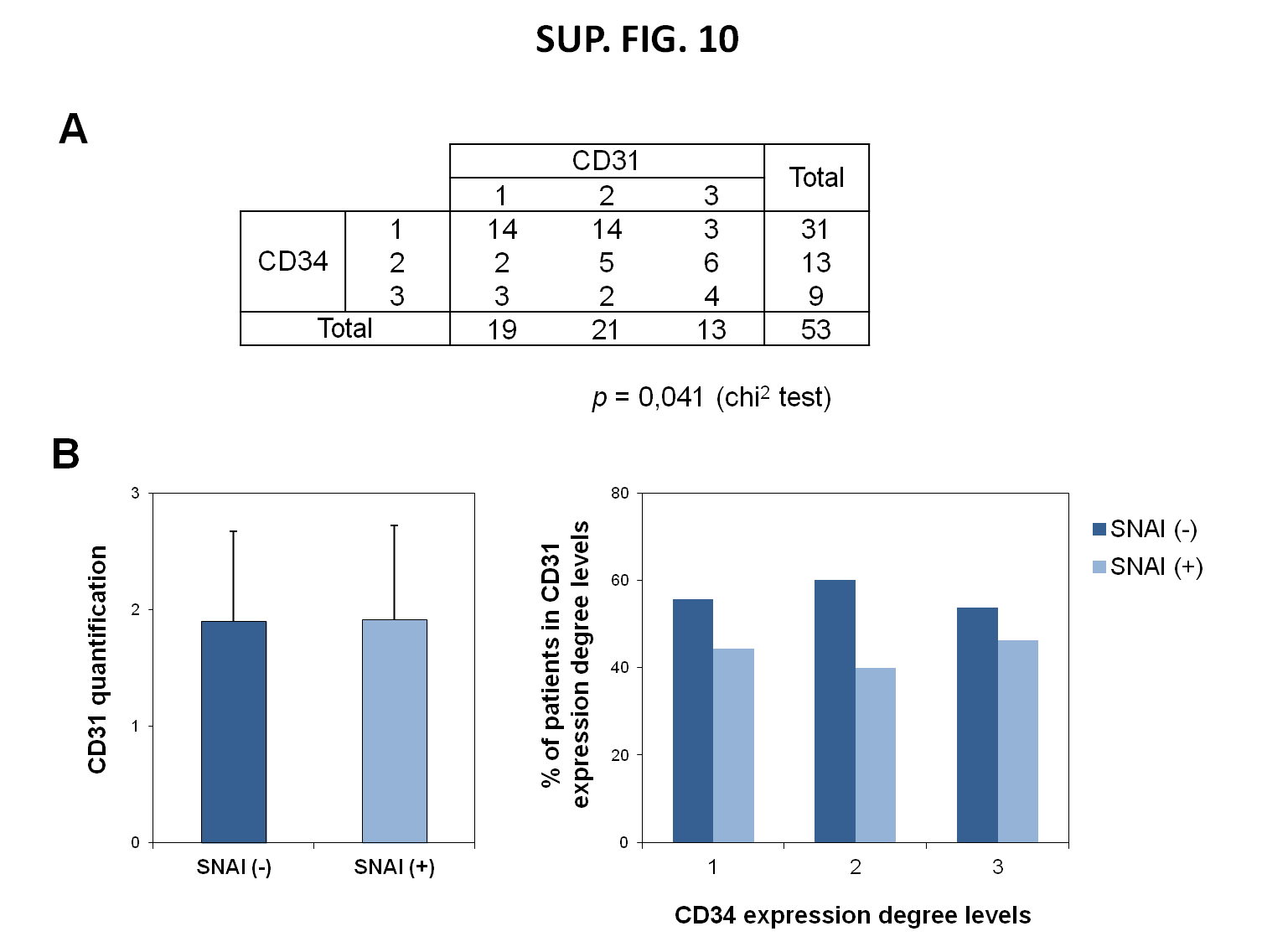

Supplement: Supplementary file 11 — Supplemental Figure 10 [file 41389_2018_85_MOESM11_ESM.tif]

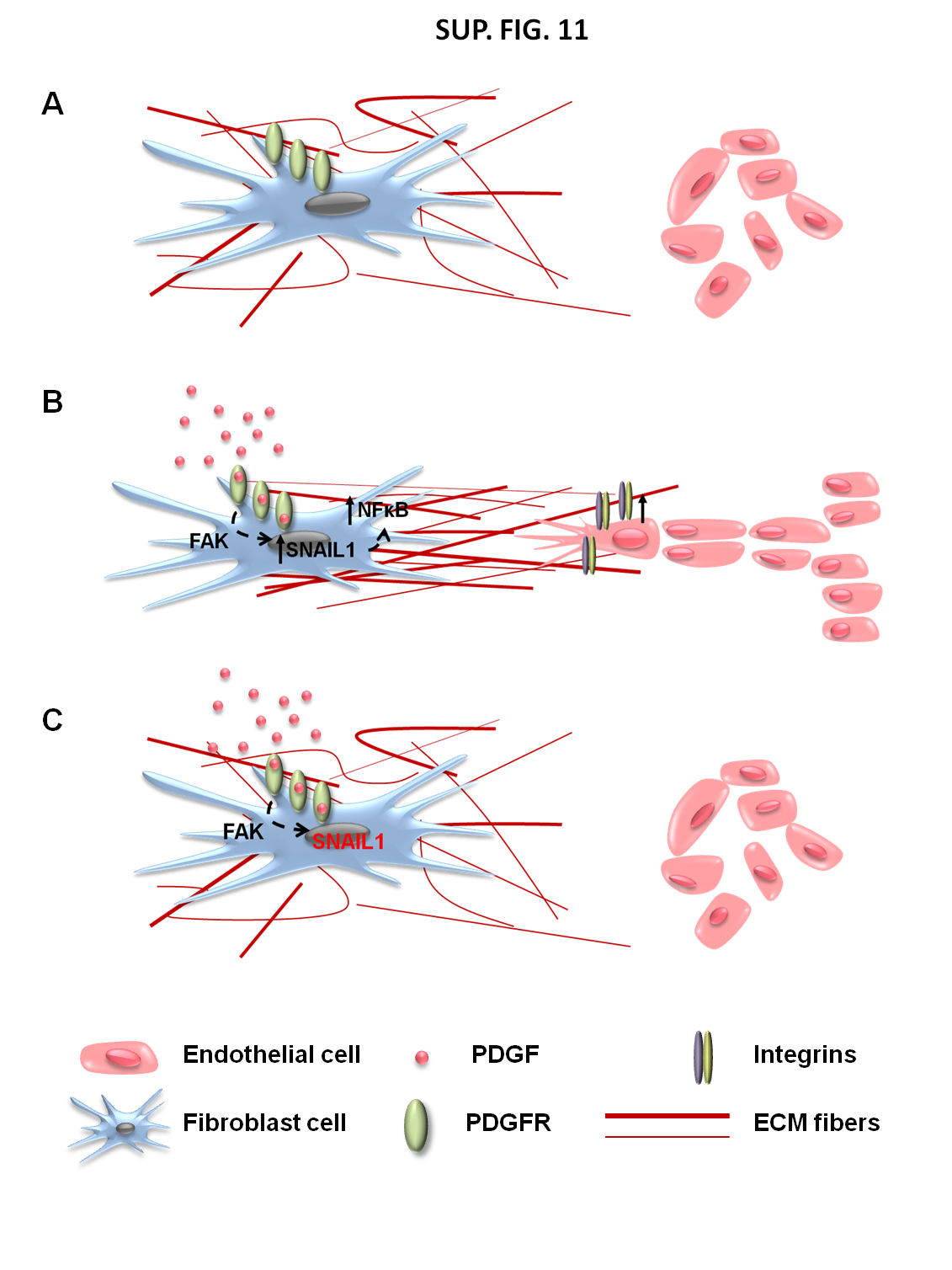

Supplement: Supplementary file 12 — Supplemental Figure 11 [file 41389_2018_85_MOESM12_ESM.tif]
